# A suite of phenotypic assays to ensure pipeline diversity when prioritizing drug-like *Cryptosporidium* growth inhibitors

**DOI:** 10.1038/s41467-019-09880-w

**Published:** 2019-04-23

**Authors:** Rajiv S. Jumani, Muhammad M. Hasan, Erin E. Stebbins, Liam Donnelly, Peter Miller, Connor Klopfer, Kovi Bessoff, Jose E. Teixeira, Melissa S. Love, Case W. McNamara, Christopher D. Huston

**Affiliations:** 1 Department of Medicine, University of Vermont Larner College of Medicine, Burlington, VT 05405 USA; 2 Cellular, Molecular and Biomedical Sciences Graduate Program, University of Vermont, Burlington, VT 05405 USA; 3 Calibr at The Scripps Research Institute, La Jolla, CA 92037 USA; 4 Department of Microbiology and Molecular Genetics, University of Vermont Larner College of Medicine, Burlington, VT 05405 USA; 5 Present Address: Novartis Institute for Tropical Diseases, Novartis Institutes for BioMedical Research, Emeryville, CA 94608 USA; 6 Present Address: Department of Surgery, Stanford University School of Medicine, Palo Alto, CA 94305-5101 USA

**Keywords:** Phenotypic screening, Parasitology, Parasitic infection, Experimental models of disease

## Abstract

Cryptosporidiosis is a leading cause of life-threatening diarrhea in children, and the only currently approved drug is ineffective in malnourished children and immunocompromised people. Large-scale phenotypic screens are ongoing to identify anticryptosporidial compounds, but optimal approaches to prioritize inhibitors and establish a mechanistically diverse drug development pipeline are unknown. Here, we present a panel of medium-throughput mode of action assays that enable testing of compounds in several stages of the *Cryptosporidium* life cycle. Phenotypic profiles are given for thirty-nine anticryptosporidials. Using a clustering algorithm, the compounds sort by phenotypic profile into distinct groups of inhibitors that are either chemical analogs (i.e. same molecular mechanism of action (MMOA)) or known to have similar MMOA. Furthermore, compounds belonging to multiple phenotypic clusters are efficacious in a chronic mouse model of cryptosporidiosis. This suite of phenotypic assays should ensure a drug development pipeline with diverse MMOA without the need to identify underlying mechanisms.

## Abstract (web summary)

Here, the authors provide a panel of medium-throughput assays to test potential drug candidates against different life cycle stages of *Cryptosporidium* with the goal to support a drug development pipeline that contains compounds with diverse molecular mechanisms of action.

## Introduction

Diarrhea causes ~8% of deaths globally in children under 5 years of age^1^. Amongst infectious etiologies, cryptosporidiosis recently garnered increased interest when the Global Enteric Multicenter Study (GEMS) reported it as a leading cause of life-threatening childhood diarrhea in Africa and the Indian subcontinent^2,3^. Cryptosporidiosis was also strongly associated with malnutrition and mortality. Infection of the intestinal epithelium by apicomplexan *Cryptosporidium* parasites causes cryptosporidiosis, and although more than twenty species of *Cryptosporidium* have been reported, *Cryptosporidium parvum* and *Cryptosporidium hominis* account for almost all human cases^4^. Despite numerous earlier studies that suggested the importance of *Cryptosporidium* parasites in young children^5^, cryptosporidiosis was previously known best as a cause of prolonged diarrhea in immunocompromised people, especially AIDS patients in whom it is reported to cause as much as 50% of chronic diarrhea^6,7^. Unfortunately, treatment options for cryptosporidiosis are very limited^4^. The only approved drug, nitazoxanide, is equivalent to a placebo in AIDS patients^8^. Nitazoxanide’s efficacy in young children is modest, with improvement after 1 week of treatment in just over half of children studied compared to spontaneous improvement of approximately one-quarter of untreated children^9^. Thus, there is a clear need for improved drugs to treat children and immunocompromised people with cryptosporidiosis.

Since there is no highly effective treatment for cryptosporidiosis in the most affected populations, it follows that there is no well-validated developmental pathway for anticryptosporidial drugs^10,11^. Early-stage investments should ideally be made in compounds with a diverse set of molecular mechanisms of action (MMOA), since there is currently no means to judge which will succeed and which will fail^12^. A pipeline of compounds with diverse MMOA is also desirable given the potential for emergence of drug resistance. Maintaining mechanistic diversity within the portfolio of compounds in development presents a technical challenge. A confounding factor is the prevalence of phenotypic screens used to identify *Cryptosporidium* growth inhibitors that work via unknown MMOA^13–16^. This problem is further compounded by the fact that, despite successful use of CRISPR/Cas9 for genome manipulation, genetic studies of *Cryptosporidium* must presently be carried out in mice^17,18^, complicating the already daunting task of drug target identification and validation.

The Medicines for Malaria Venture (MMV) employs a panel of phenotypic assays for maintenance of the malaria drug pipeline^19^. For malaria, the approach ensures the development of compounds targeting specific malaria life cycle stages, such as the liver stage, in order to address target candidate profiles for differentiated therapies (i.e., chemoprotection, treatment, and transmission blocking). The major needs for the *Cryptosporidium* field are different. As noted above, it is not even possible to ensure mechanistic diversity within the anticryptosporidial pipeline, given the landscape that the MMOA of most compounds will be unknown. In lieu of efforts for drug target identification, phenotypic assay methods are needed to maintain mechanistic diversity. To accomplish this goal, it is necessary: (1) to identify methods to distinguish compounds with different MMOA, even when their MMOA is unknown; and (2) that compounds thus separated into different groups may be efficacious in vivo (i.e., that in vivo efficacy is not correlated with any one group of compounds).

We hypothesize that orthogonal phenotypic assays will provide a means to group compounds according to different modes of action, and, while not providing specific insights into MMOA, will enable distinguishing compounds with different molecular mechanisms and thus aid in prioritizing potential anticryptosporidials. Supporting of this concept, we present moderate-throughput assays using a *C. parvum* tissue culture infection model, including assays to assess inhibition of host cell invasion, intracellular DNA replication, parasite egress and reinvasion, and sexual differentiation. By employing these mode of action assays on a diverse set of compounds, we show that the overall profiles of compounds in these phenotypic assays can be used to accurately cluster compounds into different chemical and mechanistic groups (i.e., compounds in the test set that have distinct MMOA are accurately separated based on their phenotypic profiles). And critical for the goal of using this method to maintain a mechanistically diverse developmental pipeline, we also show that compounds belonging to a variety of phenotypic clusters are efficacious in vivo. Together, these data indicate that this panel of assays can be used to maintain a diverse pipeline of anticryptosporidials, even in the absence of specific knowledge of MMOA or a validated target of different compounds/chemotypes.

## Results

### Overall strategy and anticryptosporidial compound selection

A suite of phenotypic assays roughly based on the *Cryptosporidium* life cycle was developed in order to generate phenotypic profiles of compounds. These assays were used to define a mode of action for different compounds, and then to determine if the mode of action can be used to distinguish compounds with widely divergent MMOA. For this, a test set of compounds was assembled from a variety of phenotypic screens targeting inhibition of intracellular growth during the asexual life cycle. Each of the compounds included was known to have selective toxicity for *C. parvum*. These compounds were from publicly available drug screening hits and anticryptosporidial compounds publicly disclosed in the literature (e.g., the MMV Malaria Box, and National Institutes of Health (NIH) Clinical Collection)^13,14,20^. The compound collection was further enriched by the inclusion of proprietary compounds provided by collaborators within a *Cryptosporidium* Drug Accelerator consortium that was initially established by the Bill & Melinda Gates Foundation. These compounds were most often identified as *C. parvum* inhibitors by screening libraries of compounds partially developed for other indications (e.g., treatment of malaria, trypanosomiasis, tuberculosis), and unlike most known *Cryptosporidium* growth inhibitors, many of these compounds had an associated MMOA that may inform the anticryptosporidial MMOA^15,21–25^. Thus, this test set of compounds enabled us to determine if the mode of action of different compounds (i.e., the profile of phenotypic assay results) could be used to distinguish compounds with widely different MMOAs.

**Fig. 1:**
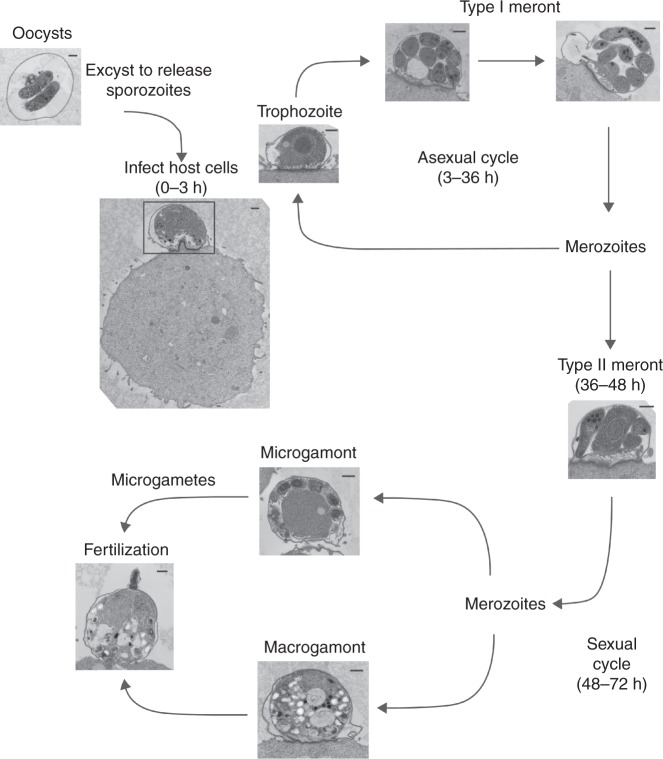
Transmission electron microscopy images showing *C. parvum* life cycle stages present in the HCT-8 cell culture system. *C. parvum* oocysts were induced to excyst and used to infect HCT-8 cell monolayers. Parasite morphology was then analyzed by transmission electron microscopy (TEM) at different time points ranging from 12 to 96 h after infection. The approximate timing of each stage is indicated. Scale bars = 500 nm. Images are representative of two independent experiments

As a first step in developing specific *Cryptosporidium* mode of action assays, we conducted a series of light microscopy time-lapse and transmission electron microscopy (TEM) time course experiments in order to precisely define *C. parvum* development within the HCT-8 cell culture system. Consistent with previously published studies^26^, all life cycle stages except oocyst formation were observed, including the development of type II meronts, macro- and micro-gametocytes, and rarely, even partial fertilization (Fig. 1). Although *Cryptosporidium* parasites in the HCT-8 cell culture system were not fully synchronized, initial excystation and cell invasion occurred within an ~2.5 h window, resulting in a roughly synchronized infection. This synchronicity was exploited by modifying a previously developed high-content microscopy assay^13^. These assay modifications enabled the evaluation of several key events within the life cycle: host cell invasion, intracellular replication, host cell egress and establishment of new parasitophorous vacuoles, and sexual differentiation. In order to determine the predominant phenotypic effect caused by a compound and enable comparison of different compounds, all compounds were tested in the assays at the 90% effective concentration (EC_90_) using a 48 h *C. parvum* growth inhibition assay (Supplementary Table 2)^13^. This concentration of each compound was expected to have its full (i.e., 90% inhibitory) effect on the most susceptible phenotype, but greater concentrations would be needed to observe the same degree of inhibition for less affected phenotypes. Since the methods all used automated liquid handling, imaging, and image analysis, the assay methods all incorporated nuclear staining to monitor host cell numbers and enable recognition of HCT-8 cell monolayer damage, for example as an artifact from a malfunctioning plate washer or from unanticipated cytotoxicity of the compounds.

### Host cell invasion assay

**Fig. 2:**
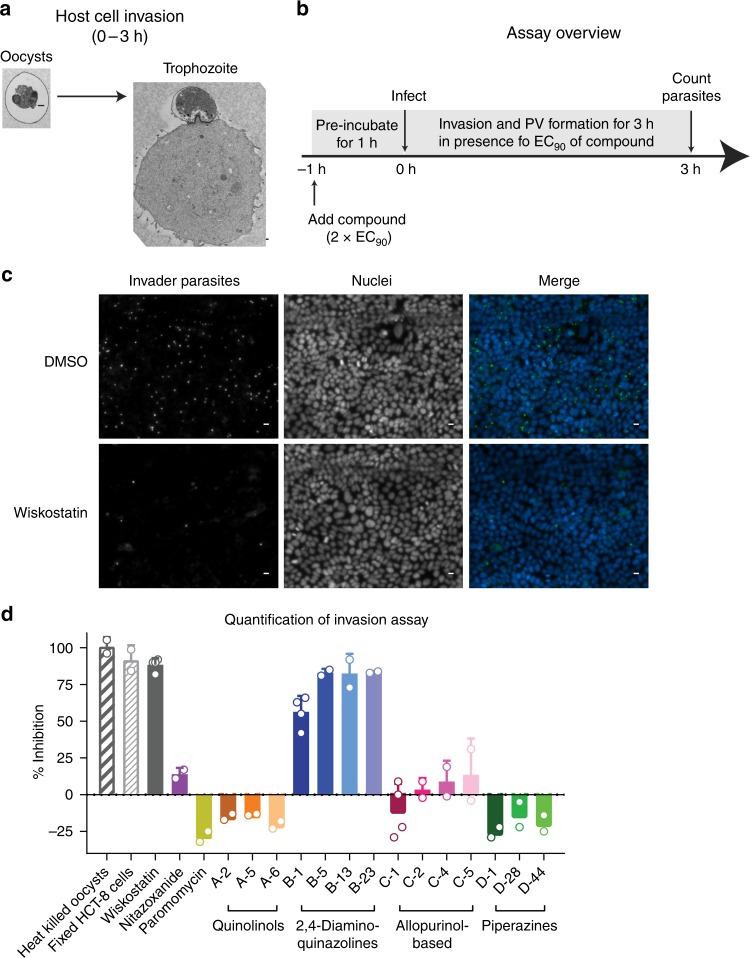
Invasion Assay. **a** Overview of life cycle stage investigated during the invasion assay. Representative TEM images of a *C. parvum* oocyst and HCT-8 cell shortly after infection (Scale bar = 500 nm). **b** Schematic of experimental design. Confluent HCT-8 monolayers were treated with 2 × EC_90_ of compounds for 1 h followed by infection with oocysts. Oocysts induced to excyst were incubated on HCT-8 cell monolayers for 3 h in the presence of EC_90_ of compounds, after which cells were washed, fixed, and parasite vacuoles were stained with a fluorescein-*Vicia villosa* lectin conjugate. **c** Representative images of parasite vacuoles (i.e., invaded parasites (green)) and host cell nuclei (blue) after treatment with DMSO control or EC_90_ (11.34 µM) of wiskostatin. Scale = 10 µm. **d** Quantified results for selected controls and compounds. The 2,4-diaminoquinazoline series (parent B-1 (MMV006169)) from the MMV Malaria Box inhibited invasion. Each point represents the mean and SD of at least two biological replicates with four technical replicates per experiment. Source data are provided as a Source Data file

The first step in infection is host cell invasion, which involves a complex process encompassing oocyst excystation, parasite motility, adhesion, and parasitophorous vacuole formation (Fig. 2a). Our strategy to assay for compounds affecting invasion was simply to expose *C. parvum* to compounds immediately after triggering excystation and throughout the invasion process, and then enumerate the parasite vacuoles after 3 h, prior to allowing the parasites to replicate (summarized in Fig. 2b). As in a *C. parvum* growth assay that we previously developed, the parasitophorous vacuoles were labeled with *Vicia villosa* lectin and detected using high-content microscopy, and the method had medium throughput and a Z’ score of ≥ 0.2^13,27^.

The Neural Wiskott-Aldrich Syndrome protein (N-WASp) and Cdc42 pathway are important for host cell actin remodeling required for *Cryptosporidium* parasitophorous vacuole formation^28^, and the small molecule wiskostatin has been shown to inhibit purified N-WASp at 10 µM, including competitive inhibition of its activation by Cdc42^29^. Therefore, wiskostatin was used as a positive control for assay validation and optimization of the *C. parvum* invasion assay. Wiskostatin was active in the 48 h *C. parvum* development assay with EC_90_ of 11.3 µM. Wiskostatin also inhibited parasitophorous vacuole formation at EC_90_ concentration. The level of non-specific binding of debris to the exterior of host cells and non-specific immunofluorescence staining was negligible, as indicated by the near complete absence of signal when measuring invasion by heat-killed parasites or invasion of fixed HCT-8 cells by viable parasites (Fig. 2c, d). As further proof-of-concept, confirmed *C. parvum* inhibitors from the MMV Malaria Box and commercially available chemical analogs of each (Supplementary Fig. 1) were tested at EC_90_ concentration measured previously for each using the 48 h *C. parvum* development assay (Fig. 2d). The chemical scaffolds tested included series of quinolinols, 2,4-diaminoquinazolines, allopurinol-based compounds, and piperazine-based compounds that were denoted as scaffold A, B, C and D, respectively (see Supplementary Fig. 1 for chemical structures). The anti-*C. parvum* 2,4-diaminoquinazolines (parent B-1 (MMV006169)) inhibited host cell invasion. In contrast, allopurinol-, quinolinol-, and piperazine-based anti-*C. parvum* compounds all had no effect on parasitophorous vacuole formation. The approved drug nitazoxanide and paromomycin, which is often used as a positive control in *Cryptosporidium* mouse models, also had no significant effect on parasitophorous vacuole formation (Fig. 2d).

### DNA replication assay

**Fig. 3:**
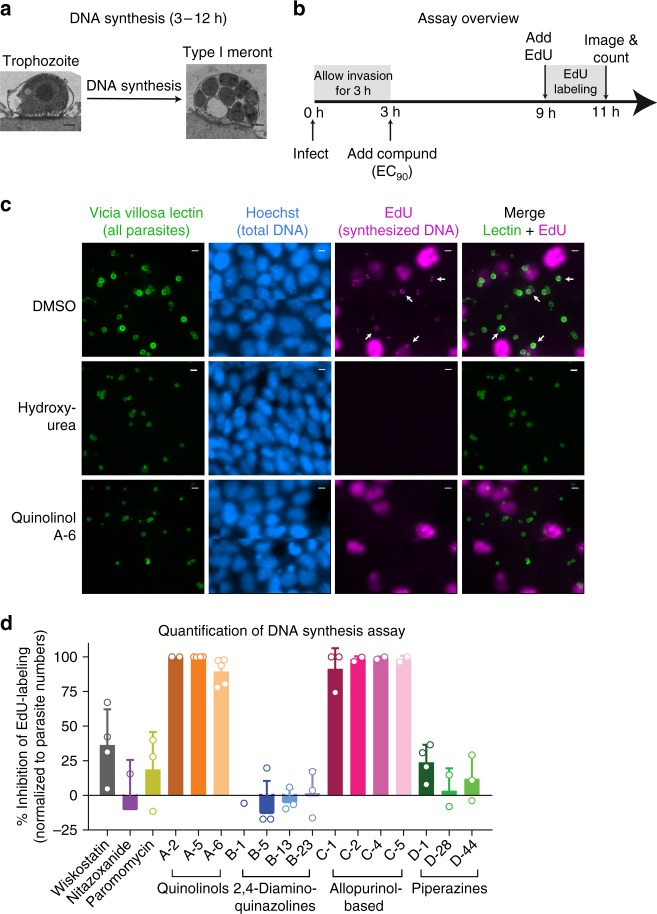
DNA Replication Assay. **a** TEM images of *C. parvum* infected HCT-8 cells demonstrating parasite morphology at the time point that the DNA replication assay is performed (Scale = 500 nm). **b** Overview of the experimental method. Confluent HCT-8 cell monolayers in glass bottom plates were infected with *C. parvum*, and compounds were added at EC_90_ following invasion. At 9 h post-infection, 10 µM EdU was added, followed by incubation for another 2 h, and then washing, fixing, and staining for microscopy. Note that images were acquired by focusing on parasite vacuoles, which typically reside in a different focal plane from the host cell nuclei. **c** Representative images of parasitophorous vacuoles (green), nuclei (blue) and EdU-labeling (magenta) after treatment with DMSO, EC_90_ of quinolinol A-6 (MMV000760) (1.33 µM), or 10 mM hydroxyurea. 40 × dry objective (NA = 0.7); scale = 5 µm. Arrows indicate selected parasites with EdU-labeled DNA. **d** Quantification of EdU incorporation. The compounds in the quinolinol and allopurinol-based series (denoted **a** and **c**, respectively) inhibited EdU incorporation. Data are mean and SD of 2–5 biological replicates. Source data are provided as a Source Data file

Following cell invasion, *Cryptosporidium* trophozoites grow and multiply to give rise to multinucleated type I meronts (Fig. 3a). The thymidine analog 5-ethynyl-2′-deoxyuridine (EdU) is efficiently incorporated into newly synthesized DNA, and can be detected using click chemistry^30^. We used EdU to measure parasite intracellular DNA replication, as a surrogate for the more complex phenotype of intracellular growth and division. The approach for differentially labeling newly synthesized parasite DNA compared to host cell DNA was to take advantage of the fact that *C. parvum* replicates more quickly than the nearly confluent host cells. Thus, by adding EdU shortly before egress, it was possible to preferentially label replicating parasites (Fig. 3b, c). DNA staining with Hoechst labeled all of the host and parasite DNA, making it challenging to visualize and quantify parasite DNA. A simple DNA stain would also not distinguish pre-existing DNA from newly synthesized DNA (Fig. 3c). In contrast, EdU labeling only occurred with incorporation into newly synthesized DNA. Incorporated EdU was then easily stained for immunofluorescence and labeled parasites were quantified using high-content microscopy and an NIH ImageJ macro (Fig. 3c, DMSO condition). The thymidine analog hydroxyurea (10 mM), a known inhibitor of DNA replication, was used as a positive control for assay validation. As expected, hydroxyurea at this concentration blocked both parasite and HCT-8 cell DNA replication (Fig. 3c). The MMV Malaria Box quinolinol A series, compounds previously identified as selective *C. parvum* inhibitors, blocked *C. parvum* DNA replication without affecting host cell DNA replication when applied at EC_90_ (Fig. 3c, d). The 2,4-diaminoquinazolines (parent B-1 (MMV006169)), which inhibited host cell invasion, had no effect on intracellular DNA replication. Results for the other compounds were also consistent within chemical groups: the allopurinol-based chemical scaffold (parent C-1 (MMV403679)) blocked *C. parvum* DNA replication, while the piperazine-based scaffold (parent D-1 (MMV665917)) and paromomycin had no effect on EdU incorporation (Fig. 3d). Compounds with activity in this assay cannot be concluded to specifically target DNA replication, which is a complex phenotype. Also, as is evident from the images, it would be feasible to enumerate the number of nuclei present in each vacuole to obtain subtler information; however, for the purpose of classifying compounds according to mode of action, we found that a readout based on *C. parvum* EdU counts normalized to total parasite numbers was readily automated using NIH ImageJ (Fig. 3d).

### Parasite egress and host cell reinvasion assay

**Fig. 4:**
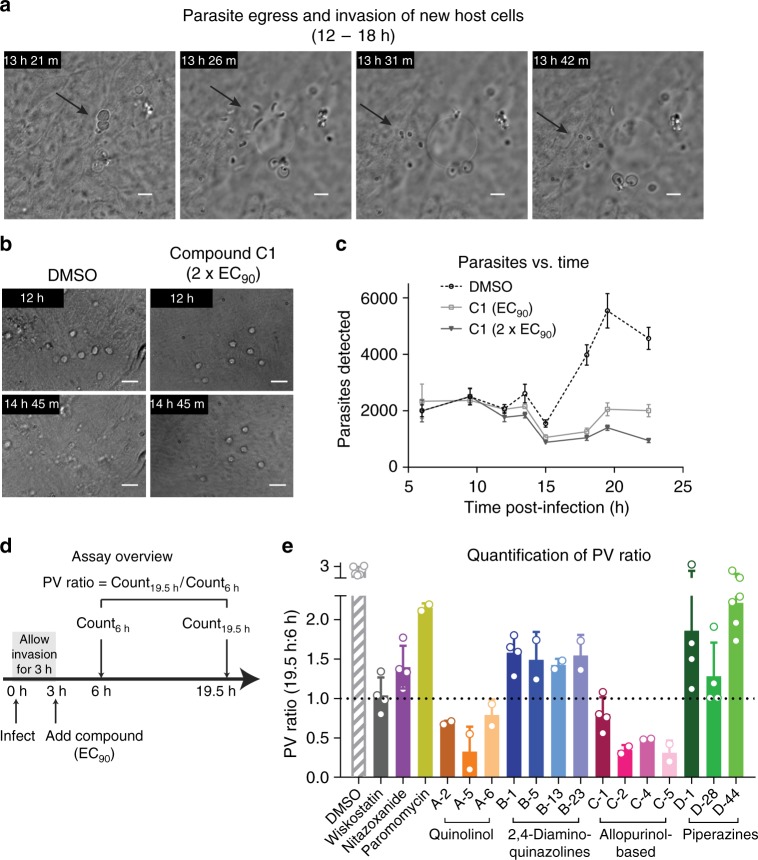
Assay to measure parasitophorous vacuole (PV) egress and invasion of new host cells. **a** Time-lapse microscopy showing the rapid events of PV egress to release motile merozoites that invade neighboring HCT-8 cells. 60 × oil objective (NA = 1.4); scale = 5 µm. **b** Time-lapse microscopy of *C. parvum* PVs in the presence of allopurinol-based compound C-1 (MMV403679) at 2 × EC_90_ (1.3 µM) or the matched DMSO control. 40 × dry objective (NA = 0.7); scale = 10 µm. **c** Time course infection experiment in the presence of DMSO or the allopurinol-based compound C-1 at EC_90_ or 2 × EC_90_. The graph shows PV numbers versus time for each condition. Data points are mean and SD, *n* = 4, representative of three independent experiments. **d** Outline of the experimental method for improved assay throughput. Infected HCT-8 cells were treated with EC_90_ of compounds 3 h after infection and PV numbers were determined at 6 h (i.e., before egress), and at 19.5 h (i.e., after egress) by immunofluorescence microscopy and an ImageJ macro. The PV ratio (count_19.5 h_/count_6 h_) is used as the assay readout. **e** Graph showing PV ratios for selected controls and the test set of compounds. All compounds reduced the PV ratio compared to DMSO, but to varying degrees and with good agreement within each chemical series. Data combined from at least two independent experiments (six for DMSO control) with four technical replicates each, mean and SD shown. Source data are provided as a Source Data file

Type I meronts release motile merozoites that infect new HCT-8 cells and repeat the asexual replication cycle. We performed live microscopy on infected cells to better understand these processes in the in vitro assay system. There was modest experiment-to-experiment variation in the timing of events, with egress observed as early as 12 h post-infection and as late as 18 h post-infection. Individual *C. parvum* egress and reinvasion events were rapid, with parasitophorous vacuole rupture, merozoite release, and reattachment completed within 10 min. Reinfection was inefficient, and each parasitophorous vacuole egress event only produced ~2–3 new vacuoles (Fig. 4a, and Supplementary Movie 1).

Compounds active in the DNA replication assay would also inhibit subsequent stages of parasite development. Nonetheless, activity in an assay of the host cell egress, migration, and cell reinvasion processes would add information by distinguishing the subset of compounds acting on these processes but with no activity at preceding life cycle stages. We used time-lapse light microscopy to assess inhibition of cell egress by the allopurinol-based scaffold (parent C-1 (MMV403679)), which was active in the DNA replication assay and therefore, expected to be useful as a positive control to aid in development of a quantitative assay for these stages of the life cycle. As anticipated, C-1 (MMV403679) at 2 × EC_90_ (1.3 µM total) inhibited parasite egress during live microscopy (Fig. 4b, and Supplementary Movies 2 and 3). Furthermore, it was clear that in this roughly synchronized culture system parasite vacuole numbers did not increase smoothly; rather, vacuole numbers increased by ratcheting up by two to three-fold during the window of egress and infection of new host cells. This observation was confirmed by comparing parasite numbers in the presence of C-1 (MMV403679) versus the vehicle control at time points approaching and just following the approximate time of cell egress and reinvasion (Fig. 4c).

This observation suggested a less labor-intensive method to estimate the effect of compounds on parasite egress and the establishment of new parasitophorous vacuoles, simply by measuring the ratio of the number of parasite vacuoles following and before the egress/reinvasion life cycle stage (Fig. 4d). Time points of 19.5 h post-infection and 6 h post-infection were selected empirically, and the parasitophorous vacuole numbers for each were then used to calculate a parasitophorous vacuole ratio (PV ratio = Count_19.5 h_/Count_6 h_). The PV ratio in six independent experiments ranged from 2.5 to 3-fold in the presence of DMSO vehicle (Fig. 4c, e). Compounds acting at all stages up to and including parasite egress and reinvasion would be expected to reduce this ratio; furthermore, rapidly parasiticidal compounds that act on early stages of the life cycle, or compounds that permit egress but block subsequent reinvasion would result in ratios below 1.

Given the complexity of this phenotype and the need to interpret the results in the context of the results of assays of earlier life stages, it is not surprising that the learner compounds tested resulted in a range of ratios. Wiskostatin, which here was added following the invasion step at 3 hours post-infection, resulted in a ratio of almost precisely one. The allopurinol-based (C) and quinolinol (A) compounds consistently displayed ratios that were under 1, whereas the piperazines (D), 2,4-diaminoquinazolines (B), nitazoxanide and paromomycin all displayed ratios above 1. As with the other assays, the results were similar for all compounds within each chemical group. And as expected, compounds acting at earlier stages showed at least some reduction in the PV ratio (Fig. 4e and Supplementary Table 1). This was especially evident for compounds such as the quinolinols and allopurinol-based compounds that blocked intracellular development, and likely occurred because no merozoites matured for egress. However, a number of compounds that had no effect in the invasion and intracellular replication assays reduced the PV ratio, indicating that this assay adds to the ability to categorize compounds (Supplementary Table 1).

### Sexual differentiation assay

Unlike malaria parasites for which only a small fraction of blood stage parasites undergo sexual differentiation, *Cryptosporidium* is believed to undergo sexual differentiation in an obligate manner after only three to four rounds of asexual replication. Furthermore, a single host serves as the site of both asexual and sexual reproduction^31^. Therefore, although all of the early-stage drug leads studied to date for cryptosporidiosis were identified using assays that measure only effects on asexual development, it is theoretically possible to target sexual development for the treatment of cryptosporidiosis. Based on this logic, we sought a method to quantify *C. parvum* sexual differentiation.

Based on the accepted *Cryptosporidium* life cycle, *C. parvum* is haploid at all stages except for just after fertilization, at which time the diploid zygote quickly undergoes meiosis to give rise to haploid sporozoites within thick and thin-walled oocysts that are either passed in the feces or excyst to perpetuate the infection^31^. Since meiosis only follows fertilization, we reasoned that genes encoding proteins specifically involved in meiosis might only be expressed in sexual forms of the parasite, including either the zygote or gamonts. Using gene expression data publicly available via CryptoDB (http://cryptodb.org)^32^, we selected the DNA Meiotic Recombinase 1 gene, DMC1 (cgd7_1690), as a likely candidate for use as a *C. parvum* sexual-stage marker, since this gene increases in expression dramatically following ~48 h of culture in HCT-8 cells and is also specific to *Plasmodium* sexual differentiation^33^. The predicted *C. parvum* DMC1 protein sequence is 99% identical to the orthologous *C. hominis* (Chro.70199) protein, and the *C. parvum* protein sequence is 65% identical to the orthologue in *Plasmodium berghei* (PBANKA_0714000).

**Fig. 5:**
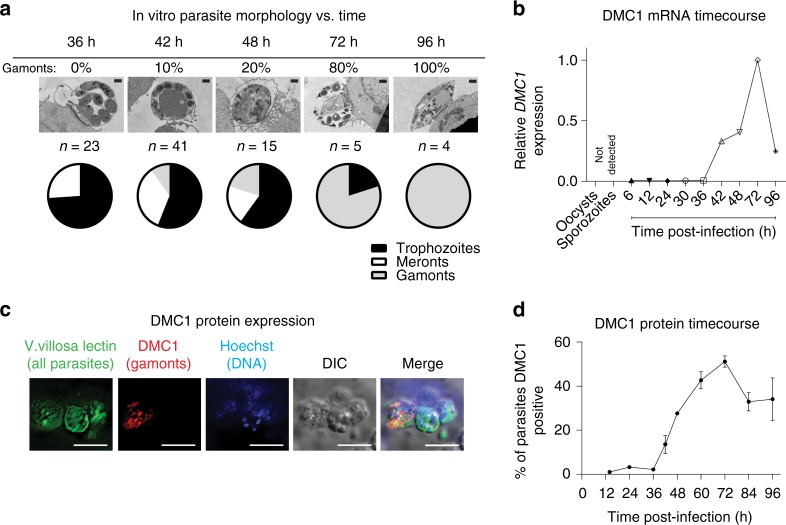
DNA Meiotic Recombinase 1 (DMC1) is a biomarker for *C. parvum* sexual development. **a** Representative transmission electron microscopy (TEM) images and results of scoring the relative abundance of different *C. parvum* life cycle stages present versus time after infection of host cell monolayers. Confluent HCT-8 cells were infected with *C. parvum* for the indicated times before preparing samples for TEM. Scale bar = 500 nm. **b** *C. parvum* DMC1 mRNA versus time during HCT-8 cell infection. Wells from the same culture plate as in **a** were used to isolate RNA, and quantitative reverse transcription PCR (qRT-PCR) was used to measure expression of *C. parvum* DMC1 (cgd7_1690) relative to 18s RNA. Data for **a** and **b** are representative of two independent experiments. **c** Immunofluorescence microscopy showing specific DMC1 expression. A mouse monoclonal anti-*C. parvum* DMC1 antibody was made and used for immunofluorescence staining and epifluorescence microscopy. DMC1 expression was limited to a subset of parasite vacuoles that contained a single nucleus. Images were acquired 72 h after HCT-8 cell infection (all parasite vacuoles (*V. Villosa* lectin staining (green)), nuclei (Hoechst (blue)), and anti-DMC1 antibody staining (red)). 60 × oil objective (NA = 1.4); scale bar = 5 µm. Images are representative of two independent experiments. **d** Time course of DMC1 protein expression. Infected HCT-8 cell monolayers grown in 384-well plates were stained as in **c** at the indicated time points. Tiled 2 × 2 images (40 × dry objective, NA = 0.7) were used to determine the percent of DMC1 parasites versus time. The graph shows mean and SD for data combined from two biological replicates. Source data are provided as a Source Data file

Real-time reverse transcription PCR (qRT-PCR) and TEM were used to determine if DMC1 expression correlated with the appearance of sexual-stage parasites in the HCT-8 culture system. To avoid inter-experimental variation in growth kinetics, samples from neighboring wells of the same infection were analyzed, focusing on finer time-points around 48 h post-infection when gamonts were first expected to appear^26^. DMC1 gene expression was first detected at 42 h post-infection, i.e., the same time gamonts were first seen by TEM (Fig. 5a, b). DMC1 mRNA levels increased by > 100-fold between 36 h and 72 h of culture, and DMC1 mRNA was not detected in sporozoites or oocysts. Interestingly, DMC1 expression decreased after peaking at 72 h post-infection.

An anti-*C. parvum* DMC1 monoclonal antibody was produced in order to determine if DMC1 protein expression also correlated with the life stages present in culture, and to generate a reagent to assay *C. parvum* sexual development by high-content immunofluorescence microscopy. A clear subset of parasite vacuoles stained positive for DMC1 at 72 h post-infection, confirming antibody specificity (Fig. 5c). DMC1 was not detected in parasitophorous vacuoles containing multiple nuclei, suggesting that it may be specifically expressed in macrogametocytes (note that fertilized zygotes are not believed to exist in the HCT-8 cell culture system). Immunogold staining and TEM with the anti-DMC1 antibody failed, however, so the specific life stage stained could not be determined with certainty. The timing of protein expression was similar to that of mRNA expression, with DMC1 positive parasites first detected at 42 h post-infection, peaking at 72 h post-infection and then decreasing in number (Fig. 5d). At peak expression levels, ~55% of the lectin-positive parasites expressed DMC1.

**Fig. 6:**
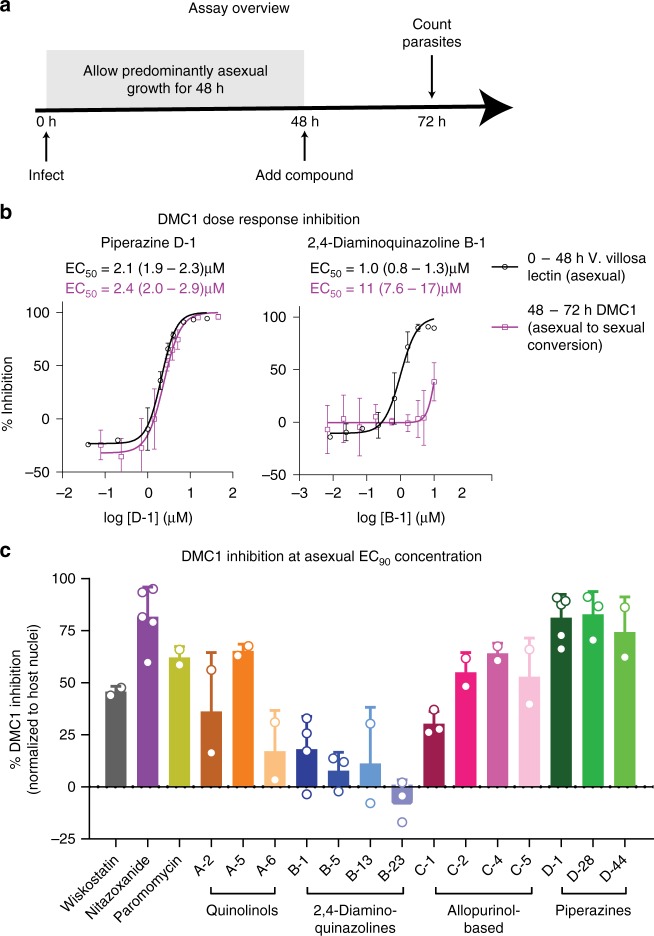
Assay to measure asexual-to-sexual-stage conversion. **a** Schematic of the experimental approach. HCT-8 cells were infected for 48 h, after which experimental compounds were added and the cultures were incubated for an additional 24 h before immunofluorescence staining with *V. villosa* lectin and anti-DMC1 antibody, and high-content microscopy. **b** Representative dose-response curves for the asexual growth assay (i.e., first 48 h) and the sexual development assay (i.e., inhibition of DMC1 expression from 48 to 72 h). Examples are shown for the piperazine compound D1 (MMV665917) and for the 2,4-diaminoquinazoline B-1 (MMV006169). **c** Graph showing the percent inhibition of DMC1 expression for control compounds and the test set of compounds. For **b** and **c** the mean and SD combined from at least two biological replicates with four technical replicates per experiment are shown. Source data are provided as a Source Data file

We utilized DMC1 as a marker of *C. parvum* sexual development, and used the anti-DMC1 monoclonal antibody to develop a moderate-throughput high-content microscopy assay for characterizing anticryptosporidial compounds (Fig. 6a). Since the percent of DMC1 positive parasites began to increase ~42 h after infection in the HCT-8 cell system, the strategy was to test the effect of compounds on sexual development simply by delaying addition of compounds until 48 h post-infection and then monitoring the effect on the number of DMC1 positive parasites at 72 h. Compounds with equal efficacy on the asexual and sexual stages would be expected to have equal potency before and after 48 h. The piperazine-based compound D-1 (MMV665917) was such a compound (Fig. 6b). In contrast, the 2,4-diaminoquinazoline B-1 (MMV006169) was less potent when used during the sexual phases of development (Fig. 6b), suggesting that it is relatively specific for asexual development. In practice, many compounds gave an intermediate phenotype with partial inhibition of DMC1 expression when added at 48 h. Therefore, in order to enable numerical comparison of the ability of compounds to block sexual development with moderate throughput, we adopted a strategy of reporting the percent inhibition of DMC1 expression by the previously determined EC_90_ of each compound in the standard asexual development assay (Fig. 6c and Supplementary Table 2).

### Categorizing inhibitors based on phenotypic profiling

Each individual mode of action assay should contribute to an overall phenotypic profile. We, therefore, aimed to develop a holistic approach to analyze the mode of action assay results that would enable us to assign a pattern of activity to each compound. If working well, such a system would distinguish compounds with substantially different MMOA, while leaving compounds with similar MMOA grouped together. Thus, even in the absence of providing direct evidence about the MMOA of any given compound or of the precise life cycle stage affected, the phenotypic profiles generated would be useful for maintaining a mechanistically diverse pipeline of anticryptosporidial compounds.

**Fig. 7:**
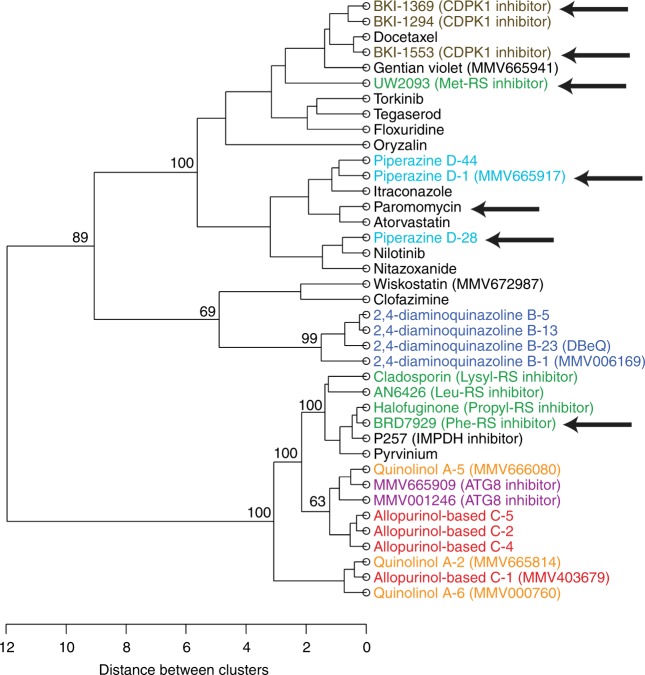
Dendrogram showing the results of a clustering analysis based on life cycle stage assay data for a diverse set of 39 *Cryptosporidium* growth inhibitors. Results from the life cycle stage assays were used to calculate a distance matrix using Euclidean distances from the mean control results, and the dendrogram was generated using the Ward error sum of squares hierarchical method. For the major nodes, bootstrap proportions ≥ 60% are shown (1000 bootstrap iterations). Singleton compounds, i.e., those with no known similarity to other compounds in the collection, are shown in black, and others are colored according to chemotype and/or putative mechanism of action. Compound structures and phenotypic assay results for each are shown in Supplementary Fig. 1 and Supplementary Table 2, respectively. NSG mouse efficacy studies performed on 31/39 compounds are summarized in Supplementary Table 3, and compounds with efficacy in the NSG mouse model at the dose tested are indicated with an arrow. Source data are in Supplementary Table 2

To test the ability to group compounds according to their overall phenotypic effects, the panel of assays described above was used to characterize a set of thirty-nine publicly disclosed compounds that were previously identified in our own screening efforts and/or provided by collaborators within the *Cryptosporidium* Drug Accelerator consortium. The compound structures and phenotypic assay data for each compound are given in Supplementary Fig. 1 and Supplementary Table 2, respectively. In addition to a number of compounds unrelated to other compounds tested, we tested numerous compounds with similar chemical structures and compounds believed to have the same or similar MMOA. We then analyzed quantitative data from each assay and compound using a clustering algorithm run in R-studio with a custom-made code (see Supplementary Method 3). One thousand bootstrap iterations were used to assess statistical rigor. The method generated a dendrogram based on the relatedness of the phenotypic effects of each compound (Fig. 7). Singleton growth inhibitors, i.e., inhibitors with no known similarity to any other test compound, are shown in black, and each additional color represents a group of compounds that is either from the same chemical scaffold (i.e., presumed to have the same MMOA) or known to have the same or a highly similar molecular target (e.g., inhibitors of several aminoacyl-tRNA sythetases (-RS inhibitors)). It is apparent from the dendrogram and bootstrapping results that related compounds clustered together, which confirmed that the overall phenotypic profile generated was adequate to distinguish compounds with substantially different MMOA.

### In vivo efficacy does not correlate with phenotypic cluster

It is feasible that *Cryptosporidium* is most susceptible to drug treatments that show the greatest activity in only one of the mode of action assays described above, which would complicate use of these assays for maintaining a diverse anticryptosporidial pipeline. Therefore, to further test the strategy of ensuring a mechanistically diverse pipeline of anticryptosporidials, it was critical to know that compounds belonging to different phenotypic effect clusters (as in Fig. 7) could work in vivo. For this, thirty-one of the thirty-nine compounds were tested in a mouse model of cryptosporidiosis^20^. There is considerable variability in the in vivo efficacy of anticryptosporidials tested in different mouse models^15,34–39^, and it remains unknown which animal models will best predict drug efficacy in humans. For example, clofazimine is efficacious in an acute, self-resolving interferon-γ knockout (IFN-γ KO) mouse model, but not efficacious in a chronic NOD SCID gamma (NSG) mouse model of cryptosporidiosis^15^. The piperazine-based compound MMV665917, however, is efficacious in both the acute IFN-γ KO and chronic NSG mouse models^40^. Most data indicate that compounds that work in the NSG mouse will also work in acute mouse models. Given that the goal of this study was to determine if compounds belonging to different phenotypic clusters might be active in vivo, we assessed in vivo efficacy using the stringent NSG mouse model. Since the current anticryptosporidial target product profile stresses efficacy superior to that of nitazoxanide, which shows no effect in the NSG mouse model, we defined in vivo efficacy for the purposes of our study as any statistically significant reduction in parasite shedding following a short, 4-day treatment course.

The mouse treatment regimens used and the effect of each on fecal *C. parvum* oocyst shedding are given in Supplementary Table 3. Compounds that were efficacious in the NSG mouse model are also indicated with arrows in Fig. 7. Note that the selection of treatment regimens was distinct for individual compounds and that often the regimen tested was selected in the absence of pharmacokinetic data, recognizing that pharmacokinetic correlates of in vivo efficacy for anticryptosporidials are not yet clearly defined. These studies, therefore, do not enable comparisons of different compounds, and were not designed for determining an optimal anticryptosporidial phenotypic profile. Rather, the goal was simply to determine if compounds belonging to the different phenotypic effect clusters could be active in vivo, which was required for the mode of action assays/compound grouping method shown above to be useful for maintaining portfolio diversity. Consistent with this possibility, there was no specific mode of action assay or phenotypic cluster that correlated with in vivo efficacy, and one or more members of multiple compound clusters were effective in the NSG mouse model.

## Discussion

Development of a drug entails significant investment following early-stage lead identification, and there is considerable attrition of compound series as they progress in development. Thus, it is ideal to begin with a diverse portfolio of drug leads, without which it is possible that multiple compounds will fail in late stages of development due to similar, previously unrecognized liabilities. Our data show that a phenotypic profiling strategy using the mode of action assays and compound clustering approach presented here can assist in establishing and maintaining a pipeline of mechanistically diverse anticryptosporidial lead compounds. Each of the assays described is performed in a 384-well tissue culture plate format using only a small quantity of compound (due to testing only at EC_90_) in conjunction with automated liquid handling, cell imaging, and image analysis. Thus, the throughput is adequate that this approach can be used for compound prioritization within the context of a high-throughput screening effort even in the absence of knowledge of each compounds’ MMOA. This conclusion is supported by data generated using a test set of compounds for which chemical-relatedness or a presumed MMOA is known, and then accurate computer-based classification of the compounds based on relatedness (e.g., ATG-8 inhibitors, CDPK1 inhibitors, and four classes of tRNA synthetase inhibitors (-RS inhibitors) were all grouped appropriately). For compounds segregated to different clusters, it is possible to conclude that their MMOAs differ substantially, even without knowledge of their MMOA. Furthermore, compounds belonging to different phenotypic clusters were efficacious in vivo, which supports the utility of this compound classification approach for establishing a mechanistically diverse anticryptosporidial pipeline.

Large scale phenotypic screening efforts are ongoing to identify *Cryptosporidium* growth inhibitors^15,16^. Thus, the phenotypic profiling methods presented here can provide much-needed tools to help prioritize compounds identified by screening. Maintaining mechanistic diversity is important during these nascent stages of anticryptosporidial drug development both because it is unknown what will best predict efficacy in the populations most in need, and in order to ensure a future pipeline that withstands the inevitable onset of drug resistance. To augment the assays presented here, we previously reported an in vitro parasite time-kill curve assay to determine the rate of *C. parvum* elimination following compound exposure^20^. In addition to ensuring further development of compounds belonging to a range of phenotypic clusters and the typical considerations of toxicity and synthetic chemistry accessibility, we therefore also envision prioritizing rapid-acting compounds over slow-acting compounds.

Although roughly based on different *Cryptosporidium* life cycle stages, these mode of action assays are not specific for different life cycle stages. For example, the use of parasite vacuole ratios at time points before and after parasite egress from and reinvasion of host cells describes the phenotypic effects of a compound, but compounds affecting all life cycle processes that occur prior to cell egress and reinvasion also affect this phenotype. The advantages of the assay are that it is a method of adequate throughput for prioritizing large numbers of compounds, and that it adds to the overall phenotypic effect profile for a given compound. And when used together, application of the suite of assays described here produces an overall phenotypic profile that can be used to distinguish compounds with substantially different MMOA. Once a diverse pipeline is established, more precise studies of the life cycle stage affected, e.g., time-lapse microscopy, and genetic studies for drug target identification may be desirable on a smaller set of leads^17^.

The test set of compounds available to establish this method was limited to thirty-nine compounds, which comprises nearly all of the lead compounds and chemical analogues of each that are currently in development for the treatment of cryptosporidiosis and can be publicly disclosed. Nonetheless, the dendrogram created from this relatively small set of compounds clearly demonstrates that these mode of action assays can be used to determine how closely anticryptosporidials are related to each other, a conclusion that was statistically supported using bootstrapping methods. Within a compound and information sharing framework such as the *Cryptosporidium* Drug Accelerator, there is a huge opportunity to expand on this theme by analyzing more compounds. Adding additional relevant assays to the profiling methods may also be of value, and would very likely improve the accuracy of the clustering algorithm results.

To compare all compounds irrespective of potency, EC_90_ concentration from the standard growth inhibition assay was used for all of the assay methods. A single EC_90_ concentration was chosen to determine the predominant effect of the compound in each phenotypic assay with a goal of comparing compounds to enable classification according to mode of action using relatively quick and inexpensive methods that require a small quantity of each compound. It is possible that at higher concentrations, compounds would be active against more than one stage, and useful information could be gleaned by testing all of the compounds at EC_50_. It may also be worth following up with dose-response curves for all of the phenotypic assays for compounds of high interest. And for a compound with an unknown target, significantly higher potency in one assay than the others might provide a clue to its MMOA that could aide in drug target identification.

To assay invasion, sporozoites are exposed to compounds for only a short time, which restricts the effect observed to the time prior to any possible intracellular replication. Since host cells are exposed to the compound for one hour prior to the addition of parasites, the design also favors identification of compounds that inhibit invasion by acting on host cells. None of the compounds active in the invasion assay were efficacious in the NSG mouse model. This could reflect the small sample size of compounds tested, and should not be interpreted as evidence that invasion inhibitors are inactive in vivo. Rather, the design of the mouse studies included here is only adequate to demonstrate that compounds with diverse phenotypic profiles (i.e., belonging to different phenotypic clusters) can be active in vivo, which is of course required if the assays are to be used to maintain pipeline diversity.

Compounds with activity against any process occurring post-invasion during parasite replication are expected to be active in the DNA replication assay. Hence, one reason that all the aminoacyl-tRNA synthetase inhibitors were potent in this assay could be that they are all lethal to the parasite before it incorporates EdU into newly synthesized DNA. N-WASp and host actin remodeling are important for intracellular *Cryptosporidium*, in addition to their role in host cell invasion^41^. It is not surprising, therefore, that wiskostatin was active in both the DNA replication and the invasion assays.

Interestingly, all compounds had some but variable activity in the egress and reinvasion assay. A ratio of less than one would signify disintegration of existing vacuoles without egress or egress without formation of new vacuoles. The 2,4-diaminoquinazolines (parent B-1 (MMV006169) were potent sporozoite invasion inhibitors but had a ratio of greater than 1, suggesting that they do not affect merozoite invasion as efficiently. Using the parasitophorous vacuole number ratio was useful to classify compounds in a higher throughput manner than was feasible using individual time course experiments, but time course experiments could be used to further investigate specific compounds. For example, a subset of compounds could be used with time-lapse microscopy to identify the specific life stage affected, e.g., merozoite release, motility, or reinvasion.

We have shown that in vitro *C. parvum DMC1* mRNA and protein expression coincides with the appearance of sexual gamont stages in HCT-8 cells. Although a strong correlation between the appearance of gamonts and DMC1 has been demonstrated, it is only correlative. At 72 h, when 80% of the parasites were gamonts (calculated from TEM images), ~55% of parasites were DMC1 positive by immunofluorescence. Additionally, DMC1 is not expressed in multinucleated cells. Microgamonts are multinucleated due to multiple microgametes per vacuole, and fertilization occurs rarely, if at all, in the HCT-8 culture system^26^. Therefore, DMC1 is not expressed in microgamonts, and must be expressed in macrogamonts. Expression of DMC1 in macrogamonts prior to fertilization might give the parasite an advantage by accelerating sporogeny and oocyst generation.

The identification of DMC1 as a sexual life stage marker makes it more feasible to study sexual development of *Cryptosporidium*. There have previously only been screening assays to identify asexual stage growth inhibitors, but with the DMC1 antibody, a high-throughput screening assay can be developed to identify sexual development inhibitors. Apart from drug candidates, the results could yield valuable tool compounds to explore *Cryptosporidium* biology and may also provide insights into the life cycle of *Cryptosporidium*, such as when the parasite becomes committed to differentiate and if the parasite is truly obligated to differentiate in vivo.

This is the first report of dose-response curves against asexual to sexual-stage conversion. It was interesting to find compounds with similar potency in the asexual stage assay and the DMC1 expression assay. Partial inhibitors are difficult to interpret, as the *C. parvum* in vitro culture system is not fully synchronized and contains a combination of asexual and sexual stages during the transition period. The DMC1 assay was designed such that compounds were added after about 20% of parasites are already gamonts. Therefore, compounds with activity against both stages would be expected to completely inhibit DMC1, whereas fast-acting compounds with activity against asexual stage parasites still present at 48 h might appear to partially affect sexual development. It is not clear if a drug needs to be active against the asexual and sexual stages of the parasite, or if activity against any one stage is sufficient. It is interesting to note that all of the compounds that were active in vivo had some activity in the DMC1 assay. However, the data set included here is too small to enable any conclusion.

In summary, we report and validate a range of medium-throughput in vitro phenotypic assays. These assays can form the basis to compare compounds according to their overall phenotypic effect profile, and provide a means to distinguish compounds based on mechanistic diversity in the absence of direct knowledge of MMOA. We believe this approach will be useful to establish and maintain mechanistic diversity within the anticryptosporidial pipeline, which is a critical need at this early-stage of *Cryptosporidium* drug development.

## Methods

### PCR primers

The following PCR primers were used in this study as described for each procedure below. Quantitative PCR amplification of DMC1 (cgd7_1690) was performed with the following primers designed using the real-time primer design tool (Integrated DNA Technologies, Coralville, Iowa): DMC1 (cgd7_1690) forward- GTTGATGGGCGGATTTGAAAG, and DMC1 (cgd7_1690) reverse- AACAGACTTTCCCATTACCTCC. Quantitative PCR amplification of *C. parvum* 18 s rRNA was performed using the following previously described primers: Cp18s forward-TAGAGATTGGAGGTTGTTCCT, and Cp18s reverse- CTCCACCAACTAAGAACGGCC^42^.

### Cell culture, and *C. parvum* excystation and infection

Human ileocecal adenocarcinoma (HCT-8) cells were purchased from ATCC (catalog# CCL-244) and cultured in ATCC modified RPMI-1640 medium (Gibco, catalog# A10491–01), supplemented with 10% heat-inactivated fetal bovine serum (Sigma-Aldrich, catalog# 12306C) and 120 U/mL penicillin and 120 μg/mL streptomycin (Gibco, catalog# 15140-122) (culture media). For dose-response assays using DMC1, amphotericin B (Sigma-Aldrich, catalog# A2942) at 0.1–0.5 μg/mL was included in the culture media. For all experiments, HCT-8 cells between passages 9 and 39 were used. *C. parvum* (Iowa isolate) oocysts were purchased from Bunchgrass Farm (Deary, ID), stored at 4 °C and used within 5 months of collection from an infected calf. To induce excystation, oocysts were first treated with 10 mM hydrochloric acid for 10 min at 37 °C, spun at 14,000×*g* for 4.5 min at room temperature, treated with 2 mM sodium taurocholate for 10 min at 16 °C, spun as before, and resuspended in culture media for infection.

### 48 h *Cryptosporidium* growth inhibition assay

A modification of a previously reported immunofluorescence assay was used to determine the EC_50_ and EC_90_ of compounds^13^. HCT-8 cells were grown to ≥ 90% confluence in 384-well plates (Corning, catalog# 353962) and infected with 5500 oocysts per well after they were induced for excystation as described above. Different concentrations of compounds were added 3 h post-infection and plates were incubated for another 45 h in a 5% CO_2_ incubator at 37 °C. Wells were then washed 3 times with PBS containing 111 mM D-galactose (PBS-D-gal), fixed with 4% paraformaldehyde (PFA) in PBS for 15 min at room temperature, permeabilized with 0.25% Triton X-100 for 10 min at 37 °C, washed 3 times with PBS with 0.1% Tween 20, and blocked with 4% bovine serum albumin (BSA) in PBS for 2 h at 37 °C or 4 °C overnight. Parasitophorous vacuoles were stained with 1.33 µg/mL of fluorescein-labeled *Vicia villosa* lectin (Vector Laboratories, catalog# FL-1231) diluted in 1% BSA in PBS with 0.1% Tween 20 for 1 h at 37 °C, followed by addition of Hoechst 33258 (AnaSpec, catalog# AS-83219) at 0.09 mM diluted in water for another 15 min at 37 °C. Wells were then washed 5 times with PBS containing 0.1% Tween 20. A Nikon Eclipse Ti2000 epifluorescence microscope with an automated stage was programmed using NIS-Elements Advanced Research software (Nikon, USA) to focus on the center of each well and take a 3 × 3 or 6 × 6 composite image using an EXi blue fluorescence microscopy camera (QImaging, Canada) with a 20 × objective (NA = 0.45). Nuclei and parasite images were separately exported as.tif files and analyzed using macros developed on the ImageJ platform (National Institutes of Health)^13^. The only modification from the published macro used to count parasites was that the lower size threshold for parasites was decreased from 16.5 to 4 pixels (1 pixel = 0.65 µm). The same microscope, camera, and software were used for all immunofluorescence assays. The parasite numbers normalized to DMSO control (% inhibition) were plotted GraphPad Prism version 7.01. EC_50_ and EC_90_ values were calculated using the included nonlinear regression analysis for dose-response (four parameters) with a top constraint set to 100.

### Host cell cytotoxicity

Each of the compounds tested was previously known to have selective toxicity for *C. parvum* and to be non-cytotoxic for HCT-8 cells at EC_90_. To further exclude the possibility of compound cytotoxicity or experimental artifacts that might be interpreted erroneously by the automated image analysis algorithms used, host cell nuclei were stained using Hoechst 33258 (AnaSpec, catalog# AS-83219) during conduct of all phenotypic assays. Wells for which the host cell monolayer was damaged (defined as those with greater than a 4 standard deviation reduction in the number of host cells vs. vehicle control wells included on each assay plate) were excluded from analysis. Since each of the compounds was known to be highly selective for *C. parvum* compared to host cells, such wells were unusual and typically found to be due to issues with liquid handling, e.g., a faulty plate washer pin.

### Invasion assay

*C. parvum* invasion inhibition was measured by modifying the 48 h growth inhibition assay described above. HCT-8 cells at > 99% confluence in 384-well plates were pre-treated with 2 × EC_90_ of compounds for 1 h before infection with parasites. In parallel, Iowa strain oocysts were excysted. To each well, 49,500 oocysts triggered for excystation were added to HCT-8 cell monolayers containing DMSO or compounds diluted to a final concentration of EC_90_ with the addition of oocysts. Assay plates were incubated at 37 °C and 5% CO_2_ for 3 h. Cells were then washed, stained, imaged and analyzed using the same protocol as described for the 48 h *Cryptosporidium* growth inhibition assay.

### DNA replication assay

Glass bottom 96- or 384-well plates (Cellvis, catalog# P96-1.5H-N and P384-1.5H-N, respectively) were coated with 50 µg/mL fibronectin (Corning, catalog# 354008) as per the manufacturer’s protocol. HCT-8 cells were grown to greater than 90% confluence in the coated glass bottom plates. Oocysts at a concentration of 55,000 per well were triggered for excystation and added to cells. After allowing 3 h for invasion, cells were treated with EC_90_ concentration of compounds for 6 h followed by addition of 10 mM 5-ethynyl-2′-deoxyuridine (EdU). After incubation of cells with EdU for 2 h, cells were washed 3 times with PBS-D-gal and then fixed with PBS containing 4% PFA. Cells were then permeabilized and stained for EdU using the Click-iT® assay kit (Thermo Fisher Scientific, catalog# C10340) as per the manufacturer’s instructions. Cells were then imaged by focusing on parasites (note that the host cell nuclei are in a different focal plane) using a 40 × dry objective (0.7 NA) and EdU, and lectin numbers quantified using ImageJ software and the custom macro in Supplementary Method 1.

### Time-lapse microscopy for visualizing live *C. parvum* egress

Glass bottom 60 mm dishes (MatTek, catalog# P35G-1.5-14-C) were coated with 50 µg/mL fibronectin (Corning, catalog# 354008) as per the manufacturer’s protocol and then HCT-8 cells were grown to > 95% confluence before infecting cells in each dish with 48 × 10^4^ oocysts triggered for excystation. Cells were then imaged live with 5 min intervals using Nomarski optics and a ×60  oil objective (1.4 NA). To assess the effect of compound C-1 on egress using live microscopy, 16-well polystyrene dishes (Greiner Bio-One™) were similarly coated with 50 µg/mL fibronectin (Corning, catalog# 354008). HCT-8 cells were grown to > 95% confluence before infection with 15 × 10^4^ oocysts triggered for excystation. After 3 h, cells were gently washed with warm complete media to remove oocyst shells before adding either compound or DMSO. Tiled 2 × 2 images were taken live at 20 min intervals using a ×40  dry objective (0.7 NA).

### Egress, motility, reinvasion assay

For quantification of parasitophorous vacuoles over time, HCT-8 cells were grown to greater than 90% confluence in 384-well plates and infected with 11,000 oocysts after triggering excystation. Compounds were added at EC_90_ to cells 3 h after infection, and cells were then washed, stained, imaged and analyzed at different time points using the same protocol as in the standard 48 h growth inhibition assay.

### *C. parvum* DMC1 (cgd7_1690) identification

The predicted protein sequence of *Plasmodium berghei* DMC1 was obtained from the free online *Plasmodium* genome database (PlasmoDB (https://plasmodb.org)), and a protein BLAST against the *C. parvum* genome was performed using the default settings in the free online *Cryptosporidium* genome database (CryptoDB (https://cryptodb.org)).

### Quantitative Real-time PCR

HCT-8 cells were seeded into 12-well culture plates (Corning) and grown to greater than 90% confluence. Oocysts at a concentration of 1.92 × 10^5^ oocysts per well were treated as above to initiate excystation and added to cells. Infected HCT-8 cells were then incubated for varying lengths of time post-infection until RNA extraction was performed.

At given time points, infected cells were trypsinized, pelleted and the RNA was extracted by homogenizing the cell pellets with TRIzol™ (Invitrogen), followed by chloroform extraction, and ethanol precipitation^43^. RNA was purified using RNeasy kits (Qiagen) according to the manufacturer’s protocol. Superscript III First-Strand Synthesis System for RT-PCR (Invitrogen) and 730 ng of RNA per sample were used to synthesize cDNA. To amplify cDNA samples, a BIO-RAD CFX96 real-time PCR machine was used with iTaq™ Universal SYBR® Green Supermix (BIO-RAD) following the manufacturer’s protocols, including melting curve analysis. The primers DMC1 (cgd7_1690) forward and DMC1 (cgd7_1690) reverse were used for DMC1 (cgd7_1690). Expression of the DMC1 (cgd7_1690) gene was normalized to *C. parvum* 18 s rRNA using the primers Cp18s forward and Cp18s reverse, and the delta CT method^42,44^.

### Electron microscopy

HCT-8 cells were grown and infected with *C. parvum* oocysts as above for real-time PCR. Infected cells were harvested at the indicated time points using trypsin, pelleted and fixed in preparation for electron microscopy. Pelleted cells were fixed for 1 h at 4 °C using half-strength Karnovsky’s fixative (1% paraformaldehyde, 2.5% glutaraldehyde in 0.05 M cacodylate buffer (pH 7.2)), embedded in 2% agarose, cross-linked with Karnovsky’s fixative, and post-fixed with 1% osmium tetroxide^45^. Samples were then dehydrated with increasing amounts of ethanol, followed by propylene oxide (PO), and then infiltrated gradually from PO into Spurr’s resin, embedded and polymerized. Semi-thin sections (1 mm^2^) were cut with glass knives on a Reichert Ultracut microtome. Ultra-thin sections (60–80 nm) were cut with a diamond knife, retrieved onto 200 mesh copper grids, contrasted with uranyl acetate (2% in 50% ethanol) and lead citrate, and examined with a JEM 1400 transmission electron microscope (JEOL USA, Inc, Peabody, Mass) operating at 80 kV.

### DMC1 immunofluorescence microscopy

Monoclonal antibody production in mice was contracted out to GenScript who generated antibodies using recombinant *C. parvum* DMC1. Several monoclonal antibodies were screened by immunofluorescence microscopy, and clone 1H10G7 (IgG2b, kappa) was selected.

Greater than 90% confluent HCT-8 cells grown in fibronectin-coated 60 mm glass bottom dishes (MatTek) were infected with ~1 × 10^6^ excysted oocysts. At given time points post-infection, infected cells were washed 3 times with PBS containing 111 mM of D-galactose, fixed for 15 min at room temperature with 4% paraformaldehyde in PBS, and permeabilized with 0.25% triton X-100 in PBS for 10 min at 37 °C. Cells were then washed 3 times with PBS and blocked at least overnight at 4 °C with 1% BSA in PBS (blocking buffer). Cells were stained with monoclonal antibody (1H10G7), either neat culture supernatant or 12.8 µg/mL of purified IgG in blocking buffer for 1 h at 37 °C, followed by 2 washes with PBS. Cells were then co-stained with a 1:500 dilution (4 µg/mL) of Alexa Fluor 568 goat anti-mouse IgG (Invitrogen, catalog# A-11004) and 1.33 µg/mL fluorescein-labeled *Vicia villosa* lectin (Vector Laboratories, catalog# FL-1231) diluted in blocking buffer for 1 h at 37 °C. Stains were then removed and 0.09 mM Hoechst 33258 (AnaSpec, catalog# AS-83219) also diluted in blocking buffer was added for another 15 min at 37 °C, followed by 2 PBS washes and imaging on a Nikon Eclipse Ti2000 epifluorescence microscope run with NIS-Elements Advanced Research software (Nikon, USA) and equipped with an EXi blue fluorescent microscopy camera (QImaging, Canada). For enumerating parasites, tiled 2 × 2 images with 15% overlapping edges were taken using a 40 × dry objective (0.7 NA), and for finer details, Z stack images were acquired using a 60 × oil objective (1.4 NA). Lectin positive cells in focus were first counted, followed by DMC1.

For compound dose-response studies, the same assay was performed in 384-well plates and imaged using a 20 × dry objective (NA = 0.45). Tiled 4 × 4 to 6 × 6 images were taken. Images were analyzed using ImageJ software and the custom macro provided in Supplementary Method  2.

### Clustering analysis

Life stage assay results were compiled into a data set of continuous variables (see Supplementary Table 2). For each assay, the data were scaled about the mean for that assay. Scaled data were used to find the Euclidean distances of each compound from other compounds and generate a distance matrix for the entire data set. The distance matrix was then used to draw a dendrogram using the Ward error sum of squares hierarchical clustering method^46^, as modified by Murtagh and Legendre^47^. A code was developed and run with R version 3.3.3^48^ using the xlsx package^49^ for data import, magrittr^50^ for data manipulation and pvclust^51^ for dendrogram validation (Supplementary Method 3). One thousand bootstrap iterations were performed using the pvclust package. Label coloring is included only for identification purposes, and had no influence on cluster composition in the generated dendrogram.

### Mouse model of chronic *C. parvum* infection

All NOD SCID gamma mouse studies were performed in compliance with all relevant ethical regulations for animal testing and research. The study received ethical approval from the University of Vermont Institutional Animal Care and Use Committee (IACUC). The University of Vermont is fully accredited by AAALAC (Animal Welfare Assurance Number: D16–00193 (A3301–01)).

The NOD SCID gamma mouse model for treatment of established cryptosporidiosis was reported previously^20^. Eighteen to twenty-four-day old male NSG mice were purchased from Jackson Laboratories, allowed to acclimatize for one week, and then infected by oral gavage of ~1 × 10^5^ oocysts. Oocyst shedding in feces was monitored by qPCR for 18s rRNA gene with the primers Cp18s forward and Cp18s reverse^42^. DNA was extracted using the Omega bio-tek’s E.Z.N.A. stool DNA kit (catalog# SKU: D4015-02) per the manufacturer’s protocol, with a modification of initially using six flash freeze and thaw cycles in liquid nitrogen to disrupt the oocysts. Different numbers of oocysts were spiked into mouse fecal samples, followed by DNA extraction and qPCR to generate a standard curve, which was run each time. Sample results were then plotted on the standard curve in order to calculate the number of oocysts shed per mg feces. Mice consistently started shedding detectable oocysts in feces by qPCR 6 days after infection. Treatment protocols were as shown in Supplementary Table 3, with all treatments given by oral gavage of compounds beginning on the 7th day after infection. Four mice were included in each experimental group, which provides a power of 80% to detect a reduction in parasite shedding equivalent to that observed with paromomycin 2000 mg/kg once daily for four days (vs. the DMSO control). Power calculations were performed using Statistical Solutions, LCC’s software. Compound efficacy was defined as a statistically significant reduction in fecal oocyst shedding compared to the vehicle control on the day following completion of treatment (one-tailed Student’s *t*-test; *p* ≤ 0.05).

### Reporting summary

Further information on research design is available in the Nature Research Reporting Summary linked to this article.

## Supplementary information

Supplementary Information

Reporting Summary

Description of Additional Supplementary Files

Supplementary Movie 1

Supplementary Movie 2

Supplementary Movie 3

Source Data

## Data Availability

All relevant data will be made available without restrictions upon inquiry. The source data underlying Figs 2, 3, 4, 5, 6, 7, and Supplementary Tables 2 and 3 are provided as a Source Data File.
