# Supplementary material for: A suite of phenotypic assays to ensure pipeline diversity when prioritizing drug-like Cryptosporidium growth inhibitors
Source: Nat Commun. 2019 Apr 23;10:1862. doi: 10.1038/s41467-019-09880-w (PMC6478823; doi:10.1038/s41467-019-09880-w)
Supplement: Supplementary file 1 — Supplementary Information [file 41467_2019_9880_MOESM1_ESM.pdf]

Supplementary Methods

Supplementary Method 1. ImageJ macro for quantification of DNA synthesis assay.

```
Title = getTitle();
Title = replace(Title, ".tif", "");
run("Stack to Images");
rename ("lectin-"+Title);
selectWindow(Title+"-0001");
rename ("edu-"+Title);
run("Enhance Local Contrast (CLAHE)", "blocksize=9 histogram=256 maximum=3
mask=*None*");
run("Enhance Local Contrast (CLAHE)", "blocksize=9 histogram=256 maximum=3
mask=*None*");
run("Subtract Background...", "rolling=1");
setAutoThreshold("Default dark");
//run("Threshold...");
setThreshold(3000, 65535);
run("Convert to Mask");
selectWindow("lectin-"+Title);
run("Subtract Background...", "rolling=15");
setAutoThreshold("Default dark");
//run("Threshold...");
setThreshold(400, 65535);
run("Convert to Mask");
run("Fill Holes");
run("Watershed");
run("Analyze Particles...", "size=50-Infinity circularity=0.50-1.00 show=[Count Masks]
display clear summarize in_situ");
setAutoThreshold("Default dark");
//run("Threshold...");
setThreshold(0, 0);
run("Create Selection");
selectWindow("edu-"+Title);
run("Restore Selection");
run("Clear", "slice");
run("Select None");
run("Analyze Particles...", "size=3-Infinity circularity=0-1.00 show=Outlines display clear
summarize in_situ");
```

Supplementary Method 2. ImageJ macro for quantification of DMC1 in the asexual to sexual conversion assay.

```
Title = getTitle();
Title = replace(Title, ".tif", "");
run("Stack to Images");
rename ("nuc-"+Title);
run("Subtract Background...", "rolling=15");
run("Unsharp Mask...", "radius=3 mask=0.70");
//run("Threshold...");
setAutoThreshold("Huang dark");
run("Convert to Mask");
run("Watershed");
run("Analyze Particles...", "size=40-Infinity show=Outlines display clear summarize in_situ");
selectWindow(Title+"-0002");
rename ("lectin-"+Title);
selectWindow(Title+"-0001");
rename ("dmc1-"+Title);
run("Subtract Background...", "rolling=1");
setAutoThreshold("Huang dark");
//run("Threshold...");
setThreshold(373, 16383);
run("Convert to Mask");
run("Fill Holes");
run("Watershed");
selectWindow("lectin-"+Title);
run("Subtract Background...", "rolling=1");
setAutoThreshold("Huang dark");
//run("Threshold...");
setThreshold(1300, 16383);
run("Convert to Mask");
run("Fill Holes");
run("Watershed");
run("Analyze Particles...", "size=2-50 circularity=0.06-1.00 show=[Count Masks] display clear
summarize in_situ");
setAutoThreshold("Huang dark");
//run("Threshold...");
setThreshold(0, 0);
run("Create Selection");
selectWindow("dmc1-"+Title);
run("Restore Selection");
run("Clear", "slice");
run("Select None");
run("Analyze Particles...", "size=0-Infinity circularity=0.5-1.00 show=Outlines display clear
summarize in_situ");
```

```
run("Images to Stack", "name=[] title=[] use");
```

Supplementary Method 3. Code used for clustering analysis using R studio.

```
#For generating Dendrograms

####### Relevent Packages and Working Directory ######
setwd("WORKING DIRECTORY") #working directory
library(xlsx)            #Importing relevant libraries
library(magrittr)
library(graphics)
library(pvclust)

####### Data Import ######

comp_df<-data.frame(read.xlsx("FILE NAME.xlsx",
                         sheetName = "Sheet1", header = TRUE, rowIndex = c(3:42),  colIndex =
c(2:9)))

names(comp_df)<-c("Name","Lab Code", "Mechanism", "ID", "Invasion", "DNAsyn",
"Motility", "SexDiff")

####### Data Cleaning ######


##Preparing Data for Dendrograms

char_list<-vector(mode = "integer", length = 33)  #Empty vector to hold count values
letter_list<-c("A","B","C","D","E","F","G","H","I","J","K",
               "L","M","N","O","P","Q","R","S","T","U","V",
               "W", "X", "Y", "Z", "AA", "AB", "AC", "AD",
               "AE", "AF", "AG") #Possible Groups, this needs additions if more groups emerge
for(x in comp_df[,3]){  #Counting group ID occurances
  char_list[which(x == letter_list)] <- char_list[which(x == letter_list)] + 1
}

repeated_values<- letter_list[which(char_list > 1)] #List of repeated groups

i<-1
for(y in comp_df[,3]){  #IDs compounds belonging to a group

  if (y %in% repeated_values){
    comp_df[i,9]<-which(as.character(repeated_values) == as.character(y))
```

```
  }else{
    comp_df[i,9]<-0
  }
  i <- i + 1
}

### Graphs Generation ###

labelColors = c("#0000FF", "#FF3030", "#228B22", "#D15FEE", "#00CED1", "#8B7355",
"#E67732", "#AD00FA", "#FEFF0E" ) #blue, red, green, orchid, turquoise, brown, purple,
bright yellow

colLab <<- function(n) {      #Function for coloring labels and assigning compund names as
labels
  if(is.leaf(n)) {
    a <- attributes(n)
    attr(n, "nodePar") <-c(a$nodePar, list(lab.col = labelColors[which(repeated_values ==
as.character(comp_df[a$label,3]))], lab.font = 2))
    attr(n, "label") <- as.character(comp_df[a$label,1])
  }
  n
}

cluster_counts<- c(2,3,4,5,6,7) #Number of Clusters

clust_df=dist(comp_df[,c(5:8)], method = "euclidean") #distance matrix with euclidean values

dendro_df<-hclust(clust_df,method = "ward.D2")%>% as.dendrogram()

####### Scaling Data Frames #########

comp_mean<-apply(na.omit(comp_df[5:8]), 2, mean)
comp_sd<-apply(na.omit(comp_df[5:8]), 2, sd)
comp_max<-apply(na.omit(comp_df[5:8]), 2, max)
comp_min<-apply(na.omit(comp_df[5:8]), 2, min)
comp_mad<-apply(na.omit(comp_df[5:8]), 2, mad)

comp_df_scaled<-data.frame(scale(na.omit(comp_df[5:8]), center = comp_mean, scale =
comp_sd))
comp_df_scaled<-cbind(na.omit(comp_df[which(!is.na(comp_df$DNAsyn)),1]),
comp_df_scaled)

clust_scaled_dist<-dist(comp_df_scaled, method = "euclidean")

dendro_scaled_dist<-hclust(clust_scaled_dist, method = "ward.D2") %>% as.dendrogram()
```

```
####### Dendrograms ############

  #margins
tiff(filename="dendrogram_coloredLabels_30Mar18.tif", width=4000, height=5000,
units="px", res=600)
par(mar = c(6,3,1,12))
plot(dendrapply(dendro_scaled_dist, colLab), horiz = TRUE,
     xlab = "Distance Between Clusters")


dev.off()
####### Using the PVClust ##############

par(mar = c(4, 4, 4, 4))  #margins

comp_df_scaled_rowNames<-comp_df_scaled
row.names(comp_df_scaled_rowNames)<-comp_df_scaled_rowNames[,1]

# PVCLUST Package Developed by Ryota Suzuki(a) and Hidetoshi Shimodaira(b)

# a) Ef-prime, Inc.

# b) Graduate School of Informatics, Kyoto University


plot(pvclust(t(comp_df_scaled_rowNames),
             method.hclust = "ward.D2",
             method.dist = "euclidean",
             iseed = 7,
             r = seq(.5, 1.4, by = .1),
             nboot = 10000),
     main = "Using the Data Set Alone \nEuclidean Distance\nWard")
```

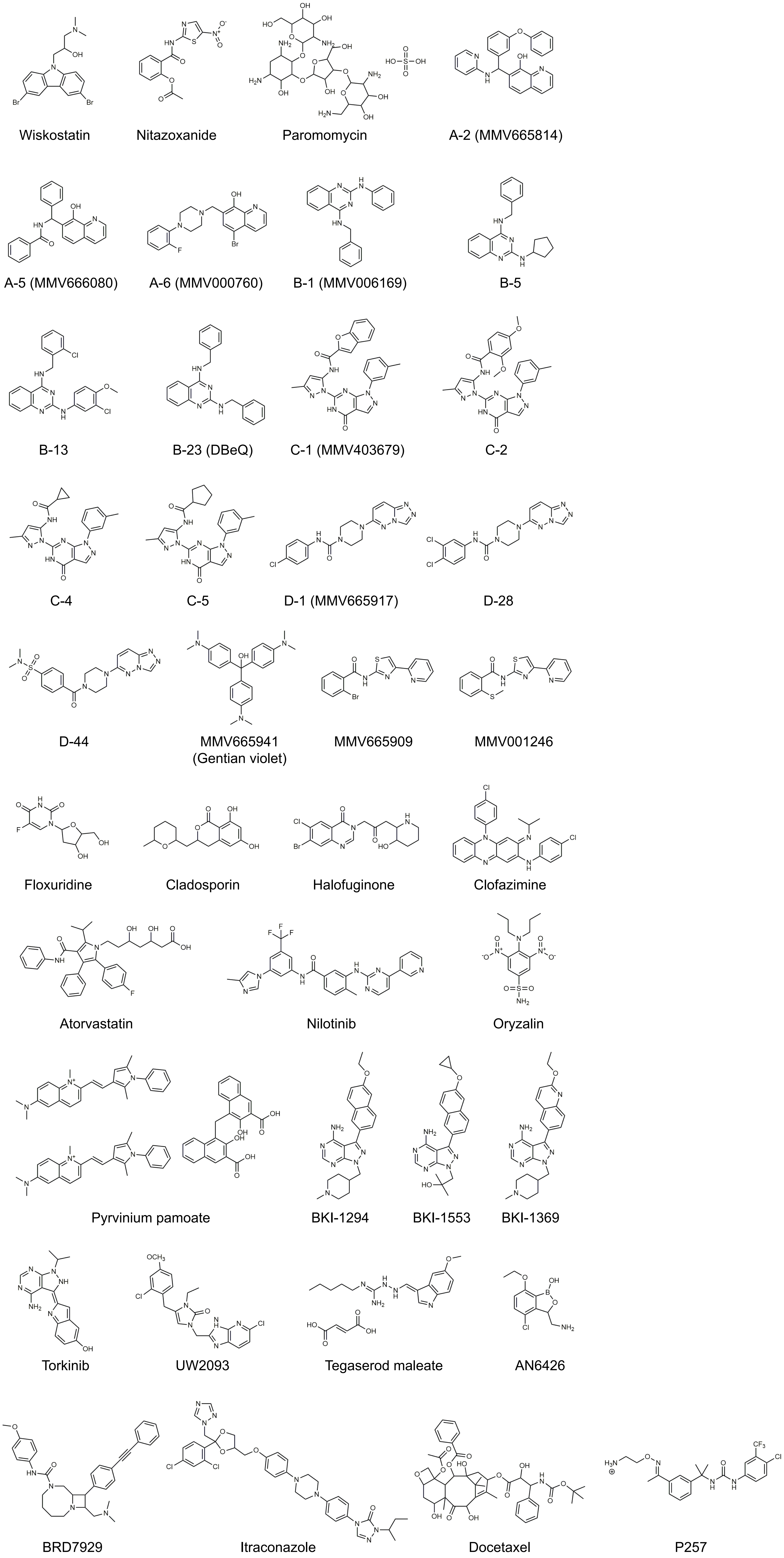

Supplementary Figure 1: Test set of compounds used for these studies. In cases where commercially available analogs were purchased (e.g. of compounds from the MMV Malaria Box), lab letter-based codes were established for each compound series. For compounds provided by collaborators, the unique identifiers are given. All of these compounds have previously been publically disclosed.

Supplementary Table 1. Examples of how several inhibitors group according to phenotypic assay results.

Scaffold class	Compound ID	Sporozoite invasion	DNA synthesis	Parasitophorous vacuole ratio (19.5 hour/6 hour)
2,4-diamino quinazolines	B-1	yes	no	1.58
	B-5	yes	no	1.49
	B-13	yes	no	1.42
	B-23	yes	no	1.55
Quinolinols	A-2	no	yes	0.69
	A-5	no	yes	0.32
	A-6	no	yes	0.79
	Floxuridine	no	no	0.74
	Tegaserod	no	no	1.01

Supplementary Table 2: Compiled assay data for all compounds. Data were generated using compounds at the EC_{90} for the asexual growth assay. Each data point represents the mean of at least 2 independent experiments.

Compound ID	Lab Code	Assigned Grouping based on putative mechanism or chemical relatedness	Regular 3 to 48 h assay *in vitro* EC_{90} (μM)	Invasion assay (mean % inhibition)	DNA synthesis (mean % inhibition)	egress and reinvasion (mean PV ratios (19.5 h:6 h))	Asexual to sexual differentiation (mean % inhibition of DMC1)	Previously published information on mechanism of action and phenotypic effects, and references.*
2,4-diaminoquinazoline B-1 (MMV006169)	B-1	D	2.27	56.43	-31.43	1.58	18.08	*C. parvum* growth inhibitor; unknown mechanism (Bessoff, et al. 2014. *AAC.*)
2,4-diaminoquinazoline B-13	B-13	D	1.39	82.51	-3.46	1.42	11.26	B-1 analog; unknown mechanism (Bessoff, et al. 2014. AAC.)
2,4-diaminoquinazoline B-23 (DBeQ)	B-23	D	10.85	83.80	1.59	1.55	-6.40	B-1 analog; unknown mechanism (Bessoff, et al. 2014. AAC.); previously reported inhibitor of mammalian p97-ATPase and the unfolded protein response (Chou, et al. 2011. PNAS.)
2,4-diaminoquinazoline B-5	B-5	D	7.22	82.99	-9.25	1.49	7.84	B-1 analog; unknown mechanism. (Bessoff, et al. 2014. AAC.)
Allopurinol-based C-1 (MMV403679)	C-1	G	0.65	-10.80	91.44	0.80	30.35	*C. parvum* growth inhibitor; unknown mechanism (Bessoff, et al. 2014. *AAC.*)
Allopurinol-based C-2	C-2	G	0.65	3.65	98.27	0.34	54.99	C-1 analog; unknown mechanism (Bessoff, et al. 2014. AAC.)
Allopurinol-based C-4	C-4	G	5.17	9.11	99.18	0.48	64.12	C-1 analog; unknown mechanism (Bessoff, et al. 2014. AAC.)
Allopurinol-based C-5	C-5	G	4.99	13.07	98.17	0.31	52.89	C-1 analog; unknown mechanism (Bessoff, et al. 2014. AAC.)
AN6426 (Leu-RS inhibitor)		H	19.51	-16.44	86.81	0.93	62.47	Leucyl-tRNA synthetase inhibitor previously reported to inhibit *C. parvum* growth (Palencia, et al. 2016. *AAC*); presumed *Cryptosporidium* protein synthesis inhibitor
Atorvastatin		P	2.18	-38.43	-4.60	1.88	36.34	Mammalian HMG-CoA reductase inhibitor; inhibits *C. parvum* growth by preventing acquisition of host cell isoprenoid precursors (Bessoff, et al. 2013. AAC)
BKI-1294 (CDPK1 inhibitor)		L	22.40	-6.64	-5.73	1.49	45.56	Calcium-dependent protein kinase (CDPK) 1 inhibitor with previously demonstrated anticryptosporidial activity in SCID beige mice and the acute IFN-γ knockout mouse infection model (Castellanos-Gonzalez, et al. 2013. *JID.* and Love, et al. 2017. *PLoS NTD.*); additional anticryptosporidial phenotypic effects not documented; BKI-1294 inhibits *Toxoplasma* and *Neospora* host cell invasion and egress (Winzer, et al. 2015. *AAC* ; Ojo et al. 2014. *PloS One.*).
BKI-1369 (CDPK1 inhibitor)		L	17.65	-7.94	16.97	1.41	41.93	BKI-1294 analog with previously demonstrated anticryptosporidial efficacy in gnotobiotic piglet model (Lee, et al. 2018. *AAC*); additional anticryptosporidial phenotypic effects not documented; BKI-1294 inhibits *Toxoplasma* and *Neospora* host cell invasion and egress (Winzer, et al. 2015. *AAC* ; Ojo et al. 2014. PloS One.)
BKI-1553 (CDPK1 inhibitor)		L	3.49	-3.92	9.91	1.94	31.88	BKI-1294 analog with *in vitro* anticryptosporidial activity; additional anticryptosporidial phenotypic effects not documented; BKI-1294 inhibits *Toxoplasma and Neospora* host cell invasion and egress (Winzer, et al. 2015. *AAC;* Ojo, et al. 2014. *PloS One.*)
BRD7929 (Phe-RS inhibitor)		H	0.15	0.40	93.39	0.72	63.11	Phenyl-tRNA synthetase inhibitor previously reported to inhibit *Plasmodium falciparum* growth (Kato, et al. 2016. *Nature*); presumed *Cryptosporidium* protein synthesis inhibitor
Cladosporin (Lysyl-RS inhibitor)		H	0.49	-6.64	93.15	0.68	94.16	Lysyl-tRNA synthetase and protein synthesis inhibitor with antimalarial activity (Hoepfner, et al. 2012. *Cell Host Microbe.*); presumed *Cryptosporidium* protein synthesis inhibitor
Clofazimine		M	19.60	66.45	88.58	0.77	89.18	Preferentially binds microbial DNA, causing cell cycle disruption; *C. parvum* growth inhibitor; active in IFN-γ knockout mouse model; inactive in NOD SCID γ mouse model (Love, et al. 2017. *PLoS NTD .)*
Docetaxel		T	1.00	-8.63	16.66	1.76	37.07	Mammalian antimitotic microtubule inhibitor; *C. parvum* growth inhibitor (Bessoff, et al. 2013. *AAC* .)
Floxuridine		J	0.14	-28.03	20.42	0.74	-7.53	Pyrimidine analog; C. parvum growth inhibitor (Bessoff, et al. 2013. AAC.)
Gentian Violet (MMV665941)		B	1.05	-6.62	47.40	1.37	34.34	Multiple reported mechanisms of action; *C. parvum* growth inhibitor (Bessoff, et al. 2014. *AAC.*)
Halofuginone (Prolyl-RS inhibitor)		H	0.34	6.92	88.20	0.69	68.25	Prolyl-tRNA synthetase inhibitor with *in vitro* anticryptosporidial activity (Jain, et al. 2017. *Structure.*); presumed *Cryptosporidium* protein synthesis inhibitor
Itraconazole		O	7.24	-29.72	-4.89	1.77	63.93	*C. parvum* growth inhibitor; unknown mechanism (Bessoff, et al. 2013. *AAC.*)
MMV001246 (ATG-8 inhibitor)		I	5.39	-7.41	72.11	0.43	45.66	Atg8-Atg3 protein-protein interaction inhibitor and *Plasmodium* growth inhibitor (Hain, et al. 2014. *J Med Chem.*); *Toxoplasma* replication inhibitor (Varberg, et al. 2018. *AAC*). *C. parvum* growth inhibitor; unknown mechanism (Bessoff, et al. 2014. *AAC.*)
MMV665909 (ATG-8 inhibitor)		I	6.84	-12.01	90.80	0.41	53.05	MMV001246 analog. Atg8-Atg3 protein-protein interaction inhibitor and *Plasmodium growth in* hibitor (Hain, et al. 2014. J Med Chem); *Toxoplasma* replication inhibitor (Varberg, et al. 2018. AAC). *C. parvum* growth inhibitor; unknown mechanism (Bessoff, et al. 2014. *AAC.*)
Nilotinib		U	16.54	-21.88	22.05	1.05	70.96	Mammalian tyrosine kinase inhibitor, including BCR-ABL, c-Kit receptor kinase, and platelet-derived growth factor receptor-beta; phenotypic screening hit; unknown anticryptosporidial mechanism.
Nitazoxanide		A	3.52	13.83	-9.16	1.40	81.79	*Trichomonas vaginalis, Entamoeba histolytica, Giardia intestinalis, Clostridium difficile, Clostridium perfringens, H. pylori,* and *Campylobacter jejuni* pyruvate-ferredoxin oxidoreductase inhibitor (Hoffman, et al. 2007. *AAC* .); current standard of care for cryptosporidiosis; anticryptosporidial phenotypic effects not documented; likely inhibits *Cryptosporidium* growth via an alternate mechanism (Bartelt, et al. 2018. *AAC.*)
Oryzalin		S	1.49	1.23	12.01	2.39	-48.24	Binds *Toxoplasma* α-Tubulin to disrupt microtubules (Morrissette, et al. 2004. *Mol Biol Cell.*); *C. parvum* growth inhibitor (Benbow, et al. 1998. *AAC.*); unknown anticryptosporidial mechanism.
P257 (IMPDH inhibitor)		V	34.30	9.88	97.76	0.84	70.52	*Cryptosporidium* inosine monophosphate dehydrogenase inhibitor; efficacious in the acute IL-12 knockout mouse model of cryptosporidiosis (Gorla, et al. 2014. *AAC.*)
Paromomycin		R	2386.00	-28.42	18.81	2.15	43.80	*C. parvum* growth inhibitor; neomycin resistance cassette shown to confer resistance (Vinayak, et al. 2015. *Nature*); active in NSG mouse model (Jumani, et al. 2018. *AAC.*)
Piperazine D-1 (MMV665917)	D-1	K	5.10	-25.16	23.98	1.86	83.01	*C. parvum* growth inhibitor; unknown mechanism. Active in NSG mouse model (Jumani, et al. 2018. *AAC.*)
Piperazine D-28	D-28	K	1.50	-13.23	3.38	1.28	81.40	D-1 analog; in vitro growth inhibitor; unknown mechanism (Jumani, et al. 2018. AAC.)
Piperazine D-44	D-44	K	11.60	-11.48	12.07	2.21	74.35	D-1 analog; in vitro growth inhibitor; unknown mechanism (Jumani, et al. 2018. AAC.)
Pyrvinium pamoate		C	3.21	25.69	88.30	0.61	68.07	Multiple reported mechanisms of action; previously demonstrated anticryptosporidial activity in neonatal mouse model (Downey, et al. 2008. *AAC.*)
Quinolinol A-2 (MMV665814)	A-2	N	1.34	-15.19	100.00	0.69	36.33	*C. parvum* growth inhibitor; unknown mechanism (Bessoff, et al. 2014. *AAC.*)
Quinolinol A-5 (MMV666080)	A-5	N	4.35	-13.46	99.95	0.32	65.35	A-2 analog. C. parvum growth inhibitor; unknown mechanism (Bessoff, et al. 2014. AAC.)
Quinolinol A-6 (MMV000760)	A-6	N	1.33	-20.21	89.49	0.79	17.22	A-2 analog. C. parvum growth inhibitor; unknown mechanism (Bessoff, et al. 2014. AAC.)
Tegaserod		F	10.32	13.93	-6.72	1.01	12.05	Mammalian serotonin Type-4 ($5-HT_4$) receptor partial agonist; *C. parvum* growth inhibitor; unknown anticryptosporidial mechanism (Bessoff, et al. 2013. *AAC.*)
Torkinib		E	0.36	-21.41	-16.16	1.61	11.19	Mammalian mTOR inhibitor; singleton phenotypic screening hit; unknown anticryptosporidial mechanism.
UW2093 (Met-RS inhibitor)		H	0.06	-9.92	83.14	1.99	7.79	Methionine-tRNA synthetase inhibitor previously reported effective for *T. brucei* in mice (Shibata, et al. 2011. *AAC.*); presumed *Cryptosporidium* protein synthesis inhibitor
Wiskostatin (MMV672987)		Q	11.34	88.58	36.39	1.04	45.76	Mammalian N-WASp inhibitor known to inhibit host cell invasion by *C. parvum* (Chen, et al. 2004. *Infect. Immun.*)

EC_{90}, indicates 90% *C. parvum* growth inhibitory concentration as measured in the regular 48 h assay (1) - citation Bessoff et al. 2013. *AAC.*

*Unless otherwise noted, anticryptosporidial phenotypic effects more specific than *in vitro* growth inhibition have not been reported.

Supplementary Table 3: Summary of *in vivo* efficacy in NOD SCID Gamma (NSG) mouse model of cryptosporidiosis.

Compound ID	Smiles	Dose (mg/kg (oral gavage))	Interval (h)	Duration (days)	Vehicle	NSG mouse efficacy	% reduction in fecal oocyst shedding vs. vehicle control [a]	p-value (student's t-test)
2,4-diaminoquinazoline B-1 (MMV006169)	C(Nc1nc(Nc2ccccc2)nc3ccccc13)c4ccccc4	50.0	12	4	1% HPMC / 5% DMSO	No	NA	
2,4-diaminoquinazoline B-13	COc1ccc(cc1Cl)Nc1nc(NCc2ccccc2Cl)c2c(n1)cccc2	50.0	12	4	1% HPMC / 5% DMSO	No	NA	
2,4-diaminoquinazoline B-23 (DBeQ)	c1ccc(cc1)CNc1nc(NCc2ccccc2)c2c(n1)cccc2	-	-	-	-	ND	NA	
2,4-diaminoquinazoline B-5	C1CCC(C1)Nc1nc(NCc2ccccc2)c2c(n1)cccc2	50.0	12	4	1% HPMC / 5% DMSO	No	NA	
Allopurinol-based C-1 (MMV403679)	c1(c(cnn1c2cccc(C)c2)C(=O)N3)N=C3n4nc(C)cc4NC(=O)c5cc(cccc6)c6o5	-	-	-	-	ND	NA	
Allopurinol-based C-2	c31c(cnn1-c2cc(ccc2)C)C(=O)NC(=N3)n4c(cc(n4)C)NC(=O)c5c(cc(cc5)OC)OC	100.0	24	7	1% HPMC / 5% DMSO	No	NA	
Allopurinol-based C-4	c31c(cnn1-c2cc(ccc2)C)C(=O)NC(=N3)n4c(cc(n4)C)NC(=O)C5CC5	100.0	24	7	1% HPMC / 5% DMSO	No	NA	
Allopurinol-based C-5	c31c(cnn1-c2cc(ccc2)C)C(=O)NC(=N3)n4c(cc(n4)C)NC(=O)C5CCCC5	100.0	24	7	1% HPMC / 5% DMSO	No	NA	
AN6426 (Leu-RS inhibitor)	OB1OC(CN)C2=C(Cl)C=CC(OCC)=C21	60.0	24	4	1% CMC/0.1% Tween	No	NA	
Atorvastatin	CC(C)C1=C(C(=C(N1CCC(CC(CC(=O)O)O)O)C2=CC=C(C=C2)F)C3=CC=CC=C3)C(=O)NC4=CC=CC=C4	50.0	12	7	1% HPMC / 5% DMSO	No	NA	
BKI-1294 (CDPK1 inhibitor)	CCOC(C=C1)=CC(C1=C2)=CC=C2C3=NN(CC4CCN(C)CC4)C5=NC=NC(N)=C53	100.0	24	4	5% DMSO 95%[7%Tween80/3%EtOH/90%Saline]	No	NA	
BKI-1369 (CDPK1 inhibitor)	CCOC(C=C1)=NC(C1=C2)=CC=C2C3=NN(CC4CCN(C)CC4)C5=NC=NC(N)=C53	60.0	24	4	90% Saline;7% Tween80;3%Etoh	Yes	90.8%	0.03
BKI-1553 (CDPK1 inhibitor)	NC1=C2C(N(CC(O)(C)C)N=C2C3=CC=C(C=C(OC4CC4)C=C5)C5=C3)=NC=N1	10.0	24	4	90% Saline;7% Tween80;3%Etoh	Yes	81.2%	0.04
BRD7929 (Phe-RS inhibitor)	CN(C)CC1C(C2N1CCCCN(C2)C(=O)NC3=CC=C(C=C3)OC)C4=CC=C(C=C4)C#CC5=CC=CC=C5	10.0	24	4	0.5% HPMC, 0.5% Tween 80/ 5% DMSO	Yes	99.9%	0.02
Cladosporin (Lysyl-RS inhibitor)	CC1CCCC(O1)CC2CC3=CC(=CC(=C3C(=O)O2)O)O	-	-	-	-	ND	NA	
Clofazimine	CC(C)N=C1C=C2C(=NC3=CC=CC=C3N2C4=CC=C(C=C4)Cl)C=C1NC5=CC=C(C=C5)Cl	100.0	24	4	Corn Oil or .5% HPMC, 0.5% Tween 80/ 5% DMSO	No	NA	
Docetaxel	CC1=C2C(C(=O)C3(C(CC4C(C3C(C(C2(C)C)(CC1OC(=O)C(C(C5=CC=CC=C5)NC(=O)OC(C)(C)C)O)O)OC(=O)C6=CC=CC=C6)(CO4)OC(=O)C)O)C)O	-	-	-	-	ND	NA	
Floxuridine	C1C(C(OC1N2C=C(C(=O)NC2=O)F)CO)O	200.0	24	7	1% HPMC / 5% DMSO	No	NA	
Gentian Violet (MMV665941)	CN(C)c1ccc(cc1)C(O)(c2ccc(cc2)N(C)C)c3ccc(cc3)N(C)C	50.0	12	4	1% HPMC / 5% DMSO	No	NA	
Halofuginone (Propyl-RS inhibitor)	C1CC(C(NC1)CC(=O)CN2C=NC3=CC(=C(C=C3C2=O)Cl)Br)O	-	-	-	-	ND	NA	
Itraconazole	CCC(C)N1C(=O)N(C=N1)C2=CC=C(C=C2)N3CCN(CC3)C4=CC=C(C=C4)OCC5COC(O5)(CN6C=NC=N6)C7=C(C=C(C=C7)Cl)Cl	50.0	12	7	1% HPMC / 5% DMSO	No	NA	
MMV001246 (ATG-8 inhibitor)	CSc1ccccc1C(=O)Nc2nc(cs2)c3ccccn3	50.0	12	4	1% HPMC / 5% DMSO	No	NA	
MMV665909 (ATG-8 inhibitor)	Brc1ccccc1C(=O)Nc2nc(cs2)c3ccccn3	50.0	12	4	1% HPMC / 5% DMSO	No	NA	
Nilotinib	Cc1ncn(c1)c1cc(NC(=O)c2ccc(C)c(Nc3nccc(n3)c3cccnc3)c2)cc(c1)C(F)(F)F	42.0	8	4	90% Saline;7% Tween80;3%Etoh	No	NA	
Nitazoxanide	CC(=O)OC1=CC=CC=C1C(=O)NC2=NC=C(S2)[N+](=O)[O-]	200.0	24	7	1% HPMC / 5% DMSO	No	NA	
Oryzalin	CCCN(CCC)C1=C(C=C(C=C1[N+](=O)[O-])S(=O)(=O)N)[N+](=O)[O-]	-	-	-	-	ND	NA	
P257 (IMPDH inhibitor)	[NH3+]CCO/N=C(C1=CC=CC(C(C)(C)NC(NC2=CC=C(Cl)C(C(F)(F)F)=C2)=O)=C1)\C	83.0	8	4	90% Saline;7% Tween80;3%Etoh	No	NA	
Paromomycin	C1C(C(C(C(C1N)OC2C(C(C(C(O2)CO)O)O)N)OC3C(C(C(O3)CO)OC4C(C(C(C(O4)CN)O)O)N)O)O)N.OS(=O)(=O)O	2000.0	24	4	1% HPMC / 5% DMSO	Yes	92.9%	0.03
Piperazine D-1 (MMV665917)	Clc1ccc(NC(=O)N2CCN(CC2)c3ccc4nncn4n3)cc1	30.0	12	4	1% HPMC / 5% DMSO	Yes	98.9%	0.01
Piperazine D-28	O=C(N1CCN(CC1)c1ccc2n(n1)cnn2)Nc1ccc(c(c1)Cl)Cl	60.0	12	4	1% HPMC / 5% DMSO	Yes	99.9%	0.01
Piperazine D-44	O=C(c1ccc(cc1)S(=O)(=O)N(C)C)N1CCN(CC1)c1ccc2n(n1)cnn2	-	-	-	-	ND	NA	
Pyrvinium pamoate	CN(C1=CC2=CC=C([N+](C)=C2C=C1)/C=C/C3=C(N(C4=CC=CC=C4)C(C)=C3)C)C.CN(C5=CC6=CC=C([N+](C)=C6C=C5)/C=C/C7=C(N(C8=CC=CC=C8)C(C)=C7)C)C.OC(C9=CC%10=CC=CC=C%10C(CC%11=C%12C=CC=CC%12=CC(C(O)=O)=C%11O)=C9O)=O	2.5	12	7	1% HPMC / 5% DMSO	No	NA	
Quinolinol A-2 (MMV665814)	Oc1c(ccc2cccnc12)C(Nc3ccccn3)c4cccc(Oc5ccccc5)c4	50.0	12	4	1% HPMC / 5% DMSO	No	NA	
Quinolinol A-5 (MMV666080)	Oc1c(ccc2cccnc12)C(NC(=O)c3ccccc3)c4ccccc4	50.0	12	4	1% HPMC / 5% DMSO	No	NA	
Quinolinol A-6 (MMV000760)	Oc1c(CN2CCN(CC2)c3ccccc3F)cc(Br)c4cccnc14	-	-	-	-	ND	NA	
Tegaserod	CCCCCN=C(N)NNC=C1C=NC2=C1C=C(C=C2)OC.C(=CC(=O)O)C(=O)O	200.0	24	7	1% HPMC / 5% DMSO	No	NA	
Torkinib	CC(C)N1C2=C(C(=C3C=C4C=C(C=CC4=N3)O)N1)C(=NC=N2)N	42.0	8	4	90% Saline;7% Tween80;3%Etoh	No	NA	
UW2093 (Met-RS inhibitor)	ClC1=CC([CH3]=O)=CC=C1CC(N2CC)=CN(CC3=NC(C=CC(Cl)=N4)=C4N3)C2=O	50.0	12	4	1%HMC 0.5% Tween80	Yes	94.6%	0.03
Wiskostatin (MMV672987)	OC(CN(C)C)CN1C2=CC=C(Br)C=C2C3=C1C=CC(Br)=C3<br>OR BrC(C=C3)=CC1=C3N(CC(O)CN(C)C)C2=C1C=C(Br)C=C2<br>OR CN(C)CC(CN1C2=C(C=C(C=C2)Br)C3=C1C=CC(=C3)Br)O	30.0	12	4	1% HPMC / 5% DMSO	No	NA	

EC$_{90}$, indicates 90% *C. parvum* growth inhibitory concentration as measured in the regular 48 h assay **(1) - citation Bessoff et al. 2013 AAC**

HPMC, hydroxypropyl methyl cellulose

ND, not done

[a] As determined by qPCR on fecal samples on the day after completing treatment.

Supplementary References:

1. Bessoff, K. et al. Identification of *Cryptosporidium parvum* active chemical series by Repurposing the open access malaria box. *Antimicrob Agents Chemother* **58**, 2731-9 (2014).
2. Chou, T.F. et al. Reversible inhibitor of p97, DBeQ, impairs both ubiquitin-dependent and autophagic protein clearance pathways. *Proceedings of the National Academy of Sciences of the United States of America* **108**, 4834-9 (2011).
3. Palencia, A. et al. *Cryptosporidium* and *Toxoplasma* Parasites Are Inhibited by a Benzoxaborole Targeting Leucyl-tRNA Synthetase. *Antimicrob Agents Chemother* **60**, 5817-27 (2016).
5. Bessoff, K., Sateriale, A., Lee, K.K. & Huston, C.D. Drug repurposing screen reveals FDA-approved inhibitors of human HMG-CoA reductase and isoprenoid synthesis that block *Cryptosporidium parvum* growth. *Antimicrob Agents Chemother* **57**, 1804-14 (2013).
6. Castellanos-Gonzalez, A. et al. A novel calcium-dependent protein kinase inhibitor as a lead compound for treating cryptosporidiosis. *J Infect Dis* **208**, 1342-8 (2013).
7. Love, M.S. et al. A high-throughput phenotypic screen identifies clofazimine as a potential treatment for cryptosporidiosis. *PLoS Negl Trop Dis* **11**, e0005373 (2017).
8. Winzer, P. et al. In Vitro and In Vivo Effects of the Bumped Kinase Inhibitor 1294 in the Related Cyst-Forming Apicomplexans *Toxoplasma gondii* and *Neospora caninum*. *Antimicrob Agents Chemother* **59**, 6361-74 (2015).
9. Ojo, K.K. et al. *Neospora caninum* calcium-dependent protein kinase 1 is an effective drug target for neosporosis therapy. *PLoS One* **9**, e92929 (2014).
11. Lee, S. et al. Therapeutic Efficacy of Bumped Kinase Inhibitor 1369 in a Pig Model of Acute Diarrhea Caused by *Cryptosporidium hominis*. *Antimicrob Agents Chemother* **62**(2018).
13. Kato, N. et al. Diversity-oriented synthesis yields novel multistage antimalarial inhibitors. *Nature* **538**, 344-349 (2016).
14. Hoepfner, D. et al. Selective and specific inhibition of the *Plasmodium falciparum* lysyl-tRNA synthetase by the fungal secondary metabolite cladosporin. *Cell Host Microbe* **11**, 654-63 (2012).
15. Jain, V. et al. Targeting Prolyl-tRNA Synthetase to Accelerate Drug Discovery against Malaria, Leishmaniasis, Toxoplasmosis, Cryptosporidiosis, and Coccidiosis. *Structure* **25**, 1495-1505 e6 (2017).
16. Hain, A.U. et al. Identification of an Atg8-Atg3 protein-protein interaction inhibitor from the medicines for Malaria Venture Malaria Box active in blood and liver stage *Plasmodium falciparum* parasites. *J Med Chem* **57**, 4521-31 (2014).
17. Varberg, J.M., LaFavers, K.A., Arrizabalaga, G. & Sullivan, W.J., Jr. Characterization of *Plasmodium* Atg3-Atg8 Interaction Inhibitors Identifies Novel Alternative Mechanisms of Action in *Toxoplasma gondii*. *Antimicrob Agents Chemother* **62**(2018).
18. Hoffman, P.S. et al. Antiparasitic drug nitazoxanide inhibits the pyruvate oxidoreductases of *Helicobacter pylori*, selected anaerobic bacteria and parasites, and *Campylobacter jejuni*. *Antimicrobial agents and chemotherapy* **51**, 868-76 (2007).
19. Bartelt, L.A. et al. Amixicile Reduces Severity of Cryptosporidiosis but Does Not Have In Vitro Activity against *Cryptosporidium*. *Antimicrob Agents Chemother* **62**(2018).
20. Morrissette, N.S., Mitra, A., Sept, D. & Sibley, L.D. Dinitroanilines bind alpha-tubulin to disrupt microtubules. *Mol Biol Cell* **15**, 1960-8 (2004).

21. Benbow, J.W., Bernberg, E.L., Korda, A. & Mead, J.R. Synthesis and evaluation of dinitroanilines for treatment of cryptosporidiosis. *Antimicrob Agents Chemother* **42**, 339-43 (1998).
22. Gorla, S.K. et al. Validation of IMP dehydrogenase inhibitors in a mouse model of cryptosporidiosis. *Antimicrob Agents Chemother* **58**, 1603-14 (2014).
23. Vinayak, S. et al. Genetic modification of the diarrhoeal pathogen *Cryptosporidium parvum*. *Nature* **523**, 477-80 (2015).
24. Jumani, R.S. et al. A novel piperazine-based drug lead for cryptosporidiosis from the Medicines for Malaria Venture open access Malaria Box. *Antimicrob Agents Chemother* **62**, e01505-01517 (2018).
25. Downey, A.S., Chong, C.R., Graczyk, T.K. & Sullivan, D.J. Efficacy of pyrvinium pamoate against *Cryptosporidium parvum* infection in vitro and in a neonatal mouse model. *Antimicrob Agents Chemother* **52**, 3106-12 (2008).
27. Shibata, S. et al. Selective inhibitors of methionyl-tRNA synthetase have potent activity against *Trypanosoma brucei* Infection in Mice. *Antimicrob Agents Chemother* **55**, 1982-9 (2011).
28. Chen, X.-M. et al. Cdc42 and the Actin-Related Protein/Neural Wiskott-Aldrich Syndrome Protein Network Mediate Cellular Invasion by *Cryptosporidium parvum*. *Infection and immunity* **72**, 3011-3021 (2004).
