# Supplementary material for: A suite of phenotypic assays to ensure pipeline diversity when prioritizing drug-like Cryptosporidium growth inhibitors
Source: Nat Commun. 2019 Apr 23;10:1862. doi: 10.1038/s41467-019-09880-w (PMC6478823; doi:10.1038/s41467-019-09880-w)
Supplement: Supplementary file 2 — Reporting Summary [file 41467_2019_9880_MOESM2_ESM.pdf]

nature research

Corresponding author(s): *Double-blind peer review submissions: write DBPR and your manuscript number here instead of author names.*

Reporting Summary

Nature Research wishes to improve the reproducibility of the work that we publish. This form provides structure for consistency and transparency in reporting. For further information on Nature Research policies, see Authors & Referees and the Editorial Policy Checklist.

Statistical parameters

When statistical analyses are reported, confirm that the following items are present in the relevant location (e.g. figure legend, table legend, main text, or Methods section).

n/a	Confirmed	
☐	☒	The exact sample size (*n*) for each experimental group/condition, given as a discrete number and unit of measurement
☐	☒	An indication of whether measurements were taken from distinct samples or whether the same sample was measured repeatedly
☐	☒	The statistical test(s) used AND whether they are one- or two-sided<br>*Only common tests should be described solely by name; describe more complex techniques in the Methods section.*
☒	☐	A description of all covariates tested
☐	☒	A description of any assumptions or corrections, such as tests of normality and adjustment for multiple comparisons
☐	☒	A full description of the statistics including central tendency (e.g. means) or other basic estimates (e.g. regression coefficient) AND variation (e.g. standard deviation) or associated estimates of uncertainty (e.g. confidence intervals)
☒	☐	For null hypothesis testing, the test statistic (e.g. *F*, *t*, *r*) with confidence intervals, effect sizes, degrees of freedom and *P* value noted<br>*Give P values as exact values whenever suitable.*
☒	☐	For Bayesian analysis, information on the choice of priors and Markov chain Monte Carlo settings
☐	☒	For hierarchical and complex designs, identification of the appropriate level for tests and full reporting of outcomes
☒	☐	Estimates of effect sizes (e.g. Cohen's *d*, Pearson's *r*), indicating how they were calculated
☐	☒	Clearly defined error bars<br>*State explicitly what error bars represent (e.g. SD, SE, CI)*

Our web collection on statistics for biologists may be useful.

Software and code

Policy information about availability of computer code

Data collection	NIS-Elements Advanced Research software (Nikon, USA).
Data analysis	GraphPad Prism version 7.04, ChemBioDraw Ultra (PerkinElmer, version14.0.0.117), ImageJ platform (National Institutes of Health, version 1.52a) with custom macros built provided in the supplementary methods section. A code was developed and run with R version 3.3.3 47 using the “xlsx” package for data import, “magrittr” for data manipulation and “pvclust” for dendrogram validation and complete code is provided in Supplemental Method 3.

For manuscripts utilizing custom algorithms or software that are central to the research but not yet described in published literature, software must be made available to editors/reviewers upon request. We strongly encourage code deposition in a community repository (e.g. GitHub). See the Nature Research guidelines for submitting code & software for further information.

Data

Policy information about availability of data

All manuscripts must include a data availability statement. This statement should provide the following information, where applicable:

- Accession codes, unique identifiers, or web links for publicly available datasets
- A list of figures that have associated raw data
- A description of any restrictions on data availability

All relevant data will be made available without restrictions upon inquiry. The source data underlying Figures 2, 3, 4, 5, 6, 7, and Supplemental Tables 2 and 3 are provided as a Source Data File.

Field-specific reporting

Please select the best fit for your research. If you are not sure, read the appropriate sections before making your selection.

☒ Life sciences ☐ Behavioural & social sciences ☐ Ecological, evolutionary & environmental sciences

For a reference copy of the document with all sections, see nature.com/authors/policies/ReportingSummary-flat.pdf

Life sciences study design

All studies must disclose on these points even when the disclosure is negative.

Sample size	Based on power calculation using Statistical Solutions, LCC's software, four mice were included in each experimental group, which provides a power of 80% to detect a reduction in parasite shedding equivalent to that observed with paromomycin 2000 mg/kg once daily for four days (vs. the DMSO control).
Data exclusions	No data were excluded from analyses.
Replication	In vitro data were complied from 2 to 6 biological replicates with 4 technical replicates per experiment in most cases as mentioned in the manuscript.<br>Efficacy studies in mice could not be replicated due to animal care guidelines by the University of Vermont Institutional Animal Care and Use Committee.
Randomization	Prior to infection mice were randomly assigned to different groups with arbitrary labels for compound dosing.
Blinding	Microscopy image acquisition and analysis were all automated to avoid any bias.<br>Investigators were blinded with labels for qPCR analysis of mouse fecal samples used to assess efficacy of compounds.

Reporting for specific materials, systems and methods

Materials & experimental systems

n/a	Involved in the study	
☒	☐	Unique biological materials
☐	☒	Antibodies
☐	☒	Eukaryotic cell lines
☒	☐	Palaeontology
☐	☒	Animals and other organisms
☒	☐	Human research participants

Methods

n/a	Involved in the study	
☒	☐	ChIP-seq
☒	☐	Flow cytometry
☒	☐	MRI-based neuroimaging

Antibodies

Antibodies used	Click-iT® EdU assay kit (Thermo Fisher Scientific, Catalog# C10340) was used as per the manufacturer's instructions.<br>Fluorescein-labeled Vicia villosa lectin (Vector Laboratories, Catalog# FL-1231) at 1.33 µg/mL.<br>DMC1 monoclonal antibodies were made by GenScript in accordance with their proprietary protocols. Briefly, recombinant Cryptosporidium parvum DMC1 protein with C-terminal His tag was expressed in E. coli BL21 (DE3) cells using E3 expression vector. BALB/c mice were challenged with purified recombinant protein followed by fusion of B cells with myyeloma SP 2/0 cells to produce hydridomas. Supernatants from cloned cell lines that were positive by indirect ELISA were screened for DMC1

	staining in parasite culture using fluorescence microscopy. Cell line 1H10G7 clone was selected based on specific immunoflourescence staining and culture superantants were further used for further assays.
Validation	Click-iT® EdU assay kit (Thermo Fisher Scientific, Cat# C10340) has been validated on mammalian cells (Salic A., et al. 2008 PNA). The kit was further validated in the paper for mammalian and parasite cells with data shown in Figure 3.<br>Vicia villosa lectin (Vector Laboratories, Catalog# FL-1231) at 1.33 µg/mL has been validated for detected Cryptosporidium paprasite by Bessoff K., et al. 2014, Jumani RS, et. al. 2018 among others and also throughout in this paper.<br>DMC1 was validation studies are shown in the manuscript with more data available from authors upon reasonable request.

Eukaryotic cell lines

Policy information about cell lines

Cell line source(s)	HCT-8 [HRT-18] were purchased from ATCC® (Catalog# CCL-244).
Authentication	None of the cell lines used were authenticated.
Mycoplasma contamination	The cell lines were not tested for mycoplasma contamination.
Commonly misidentified lines<br>(See ICLAC register)	Misidentified cell lines were not used in the study.

Animals and other organisms

Policy information about studies involving animals; ARRIVE guidelines recommended for reporting animal research

Laboratory animals	NOD.Cg-Prkdcscid Il2rgtm1Wjl/SzJ strain # 005557 "NOD Scid gamma (NSG)". Three week old (± 3 days) male NSG mice were purchased from Jackson Laboratories, allowed to acclimatize for one week, and then used for the study.
Wild animals	The study did not involve wild animals.
Field-collected samples	The study did not involve samples collected from the field.
