# Supplementary material for: A suite of phenotypic assays to ensure pipeline diversity when prioritizing drug-like Cryptosporidium growth inhibitors
Source: Nat Commun. 2019 Apr 23;10:1862. doi: 10.1038/s41467-019-09880-w (PMC6478823; doi:10.1038/s41467-019-09880-w)
Supplement: Supplementary file 3 — Description of Additional Supplementary Files [file 41467_2019_9880_MOESM3_ESM.pdf]

Description of Additional Supplementary Files

File Name: Supplementary Movie 1
Description: Time-lapse light microscopy showing parasitophorous vacuole rupture with egress of C. parvum and formation of new parasitophorous vacuoles.

File Name: Supplementary Movie 2
Description: Time-lapse light microscopy showing the effect of DMSO (negative control) on rupture and formation of C. parvum parasitophorous vacuoles vs. time. Several vacuoles are observed rupturing, with formation of 2-3 smaller vacuoles nearby.

File Name: Supplementary Movie 3
Description: Time-lapse light microscopy showing the effect of compound C-1 (positive control) on rupture and formation of C. parvum parasitophorous vacuoles vs. time. No vacuole rupture or new vacuole formation is observed in the presence of C-1.
